# Molecular Analysis of Selected Resistance Determinants in Diarrheal Fecal Samples Collected From Kolkata, India Reveals an Abundance of Resistance Genes and the Potential Role of the Microbiota in Its Dissemination

**DOI:** 10.3389/fpubh.2020.00061

**Published:** 2020-03-11

**Authors:** Rituparna De, Asish Kumar Mukhopadhyay, Shanta Dutta

**Affiliations:** Division of Bacteriology, National Institute of Cholera and Enteric Diseases, Kolkata, India

**Keywords:** antimicrobial resistance, antimicrobial resistance genes, microbiota, microbiome, metagenomics, diarrhea, pathogen, commensal

## Abstract

Twenty-five diarrheal fecal samples from Kolkata were examined to determine the relative abundance of antimicrobial resistance genes (ARGs) against eight common classes of antibiotics with polymerase chain reaction (PCR) and Sanger sequencing. Relative abundance of an ARG was calculated as the percentage of fecal samples showing the presence of that particular ARG. The frequency of occurrence of resistance marker against each class of antibiotic was calculated as the percentage of fecal samples carrying at least one resistance marker for that particular class of antimicrobials. Antibiogram of *Vibrio cholerae* (*V. cholerae*) O1 strains isolated from four of these samples was obtained by disc diffusion method and was compared with the ARG profile of corresponding fecal samples from which the strains were isolated. A 464 bp amplicon of the V3-V4 region of bacterial 16S rDNA was obtained by PCR from 9 of these 25 samples using the primer pair *S-D-Bact-0341-b-S-17* and *S-D-Bact-0785-a-A-21* and sequenced to determine the major operational taxonomic unit (OTU). These 9 samples represented diarrhea due to diverse etiology and also unresolved etiology as determined by culture method. We conclude that the diarrheal intestinal microbiome has a common gene pool of ARGs against the major classes of antibiotics and may be serving as a reservoir of ARG dissemination. ARG profile of cholera stool showed that ARGs present in the gut of cholera patients may be transferred to the *V. cholerae* genome and pose a serious threat to the treatment of cholera by triggering resistance against potential drugs to which contemporary strains of *V. cholerae* were found to be sensitive in the present study. Fecal samples which were culture negative for diarrheal pathogens we tested also carried ARGs and OTU. Abundance of resistance markers against macrolides, tetracyclines, and aminoglycosides was the highest. Phylum Proteobacteria was the most abundant OTU suggesting proteobacterial blooms characteristic of disturbed gut microflora. Our study is the first comparative study of ARG profile of diarrheal samples with varying etiologic agent revealing the presence of ARGs against the most important classes of antibiotics in the gut of diarrheal patients by common, robust molecular methods, which are easily accessible by molecular epidemiological laboratories worldwide.

## Introduction

Antimicrobial resistance (AMR) among diarrheal pathogens has emerged as a critical threat to the clinical management of diarrheal cases. Oral rehydration therapy (ORT) is the primary treatment for diarrhea and antibiotic therapy is used as a supplementary treatment to reduce severity and morbidity. Pathogens have developed resistance to multiple antibiotics which were used for controlling these infections giving rise to multi-drug resistance (MDR) which is leading to higher number of deaths. These are extremely difficult to treat with known chemotherapeutic agents in diarrheal patients for whom primary treatment with oral rehydration solution (ORS) is insufficient.

Common enteric pathogens like *Klebsiella pneumoniae* and *Eschericia coli* have developed resistance against last resort antimicrobials like carbapenem and these are, in turn, serving as potential agents for transmission of ARGs into the environment and the community^1^ (1). Resistome analysis to understand the antimicrobial resistance (AMR) profile in pathogens is urgently required in order to discern divisive methods to prevent the transmission and spread of genetic determinants of AMR. The members of the microbiota in the environment and in humans are the primary sources of ARGs. These serve as potential reservoirs for the persistence and transmission of ARGs. The indiscriminate use of antibiotics in farm and for clinical and veterinary practices has led to the emergence of antimicrobial resistance as a critical threat. In this study we have attempted to report about the profile of selected resistance determinants obtained from fecal samples using simple polymerase chain reaction (PCR) with specific primers to detect a spectrum of antimicrobial resistance determinants that are involved in diverse antimicrobial resistance mechanisms in bacteria and which are associated with the most common classes of antibiotics advocated for diarrheal treatment. These include tetracyclines, macrolides, amphenicol, aminoglycosides, carbapenem, trimethoprim, sulfamethoxazole, and quinolones. The antimicrobial genetic determinants selected to serve as markers for these classes of antimicrobial resistance include those encoding enzymes, efflux proteins, and proteins which are involved in diverse mechanisms like cell wall degradation of bacteria and inhibition of protein synthesis. The presence of these genetic determinants was further confirmed by sequencing these genes where standard positive control DNA was not available. The results revealed the existence of antimicrobial resistance determinants against major classes of antibiotics in fecal DNA samples. The study helped us to forebode the possibility of ARGs being transmitted in the near future from the microbiota into pathogens. Our study suggests that major members of the gut community are serving as reservoirs of ARGs as samples from which pathogens could not be isolated by conventional culture methods also presented an ARG profile. We also report the relative abundance of different ARGs in the diarrheal gut microbiota in Kolkata and the suburban areas. Our study is the first addressing the relative abundance of ARGs of different classes of antimicrobials with the help of common and economic laboratory tools in the gut microbiome of the local diarrheal patients in Kolkata and the suburban areas. The study would provide valuable understanding about the threat posed by the presence of ARGs in the gut of diarrheal patients in parts of the world where the economically backward population is under perpetual threat of diarrheal diseases due to lack of sanitation and for whom antimicrobial therapy is of utmost importance to reduce severity and mortality due to diarrhea along with ORS administration. Therefore, understanding the distribution of ARGs is important to reduce their transmission from their reservoir with interceptive methods.

## Materials and Methods

### Sample Collection and Ethical Clearance

Twenty-five diarrheal stool samples were collected from the Bacteriology Division laboratory of National Institute of Cholera and Enteric Diseases (NICED), which routinely receives stool samples from the adjoining Infectious Diseases Hospital (IDH) and the B. C. Roy Hospital (BCH) for systematic screening and isolation of enteric pathogens from stool of diarrheal patients from Kolkata and the suburban areas. Thus, these 25 samples represented diverse etiology and the diarrheal population of Kolkata and the suburbs. These patients eliminated loose watery stool more than three times in a day and suffered mild to severe dehydration. Stool samples included in the study were from patients of age 2 months and above and were from male and female patients. Twenty-four of these samples were from patients who were admitted to IDH for 1–4 days for treatment of acute diarrhea and in these patients diarrhea lasted for 1–5 days. One sample KOL18B2-6 was collected at the outpatient ward of BCH from the patient who had symptoms of mild diarrhea. In this patient diarrhea lasted for 2 days. After collection, each stool sample was given a unique identity number for the study. All stool samples were collected and handled in a manner conforming to ethical rules and regulations of the local governing bodies and the institute (NICED). Table 1 presents a description of the stool samples used for the study.

**Table 1:** Stool samples used in the study, their description and AMR profile.

**Stool sample**	**Description**	Age of patient; Sex; Location^*^	**AMR profile of fecal sample by ARG profile**	**Pathogen isolated**
KOL18B2-1	Greenish yellow liquid	29y;M;S	*tetAB,cat1,sul2,ant,dfrA12,aac(3),aac(6′),aadA1,strAB,mefA,mphA,tnpA,int1,int4*	*VC O1 Inaba*
KOL18B2-2	Off-white liquid	16y;M;K	*tetABM,cat1,floR,sul2,dhfr1,ant,dfrA12,dfr1,aac(3),aac(6′),aadA1,strAB,* *mphA,tnpA,int1,int2,int4,sxt*	*VC O1 Ogawa+C.jejuni*
KOL18B2-3	Pale yellow liquid	*55y;M;K*	*tetABCDEM,cat1,floR,sul2,ant,dfrA12,dfrA15,dfr1,aac(3),aac(6′),aadA1,strAB,* *mphA,mefA,tnpA,int1int2,int4,sxt*	*TCBS(Y)OX + String-PCR-*
KOL18B2-4	White liquid	*23;M;S*	*tetABM,cat1,sul2,ant,dfrA12,aac(3),aac(6′),aadA1,strAB,aph,mefA,* *mphA,tnpA,int1,int4*	*VC O1 Inaba+Campylobacter sp*.
KOL18B2-5	Bloody liquid	*22y;F;K*	*tetABM,sul2,ant,aac(3),aac(6′),aadA1,strAB,aph,mefA,mphA,tnpA,int1,int4*	*VC Non-O1 nonO139*
KOL18B2-6	Gray liquid	*3.6y;F,S*	*tetABM,sul2,aadA1,strAB,mefA,mphA*	No pathogen
KOL18B2-7	Green liquid	*60y;M;K*	*tetABDM,sul2,dfr1,ant,aac(3),aac(6′),aadA1,strAB,mefA,mphA,tnpA,int1,int2*	No pathogen(MAC LF)
KOL18B3-1	Off-white liquid	*35y;F;K*	*tetABM,cat1,floR,ant,dfrA12,dfr1,aac(3),aac(6′),aadA1,strAB,mphA,* *mefA,tnpA,int1int2,int4,sxt*,	*VC O1 Ogawa*
KOL18B3-2	Greenish yellow liquid	*70y;M;K*	*tetABM,cat1,ant,dfrA12,aac(6′),aadA1,strAB,mphA,mefA,int1,int4*	*No pathogen*
KOL18B3-3	Whitish-green liquid	*1y;M;S*	*tetEM,ant,aadA1,strAB,mphA,mefA,tnpA,int1*	*EAEC*
KOL18B3-4	Bloody, mucoid, liquid	*4y;M;K*	*tetAB,cat1,sul2,ant,dfr1,aac(3),aac(6′),aadA1,strAB,mefA,mphA,tnpA,int1,int2*	*HEA (Green)TSI(k/A+G)*
KOL18B3-5	Brown liquid	*55y;F;S*	*tetABM,sul2,dfr1,aac(6′),aadA1,strAB,mefA,mphA,tnpA,int1*,	*S.flexeneri2a,C.coli*
KOL18B3-6	Brown liquid	*65y;M,K*	*tetAB,cat1,sul2,ant,dfr1,aadA1,strAB,mefA,mphA,tnpA,int1,int2*	*S.flexeneri 2a*
KOL18B3-7	Yellowish liquid	*25y;M;K*	*tetABM,dfr1,aac(3),strAB,mefA,mphA,tnpA,int2*	No pathogen(MAC LF)
KOL18B3-8	Bloody, mucoid, liquid	*53y;M;S*	*tetAB,dfr1,sul2,aac(3),strAB,mefA,mphA,int2*	*Aeromonas sp*.
KOL18B3-9	Greenish yellow liquid	*65y;M,K*	*tetABM,sul2,ant,dfrA12,aac(3),aac(6′),aadA1,strAB,mefA,mphA,tnpA*	*HEA (Green)TSI(k/A+G)*
KOL18B3-10	Bloody liquid	*30y;F;K*	*tetABM,sul2,strAB,mefA,mphA,tnpA*	*Mac (NLF)TSI(k/A+G)*
KOL18B3-11	Yellowish liquid	*9m;M;S*	*tetM,strAB,mefA,mphA*	*Aeromonas sp*.
KOL18B3-12	Gray liquid	*50y;F;K*	*tetABDEM,sul2,ant,dfr1,aph,aac(3),aadA1,strAB,mefA,mphA,tnpA*	*Aeromonas sp*.
KOL18B3-13	Transparent liquid	*2m;M;S*	*dfr1,mefA,int2*	*S.sonnei*
KOL18B3-14	Greenish white semi-solid	*7m;M;S*	*tetA,ant,dfr1,aac6,strAB,mefA,mphA,tnpAint2*	*EAEC*
KOL18B3-15	Bloody, mucoid semi-solid	*40y;M;K*	*tetABM,cat1,sul2,ant,dfr1,aac(3),aac(6′),aadA1,strAB,mefA,mphA,tnpA,int2,int4*	*S.flexeneri*
KOL18B3-16	Brown liquid	*45y;F;K*	*tetA,sul2,aac(3),aac(6′),strAB,mefA,mphA,tnpA*,	No pathogen(MAC LF/NLF)
KOL18B3-17	White liquid	*7m;M;K*	*tetABM,sul2,aac(3),aac(6′),strAB,mefA,mphA,tnpA*,	No pathogen(MAC LF/NLF)
KOL18B3-18	Off-white mucoid liquid	*7y;M;S*	*tetABM,sul2,ant,dfr1,aac(3),aac(6′),strAB,mefA,mphA,tnpA*,	*S.flexeneri*

Location

* *: K, Kolkata; S, Suburban area*.

*Age: y, years; m, months*.

*Sex: M, Male; F, Female*.

### Isolation of Genomic DNA From Standard Laboratory Strains

Standard laboratory strains N16961 (*V. cholerae* O1, El Tor), O395 *V. cholerae* O1, classical) and MO10 (*V. cholerae* O139) served as control for this study. The strains were obtained from the NICED strain repository and initially grown overnight at 37°C on TCBS (thiosulphate- citrate- bile salts-sucrose) (BD Difco™, U.S.A) plates followed by sub-culturing on Luria agar (BD Difco™, U.S.A) plates containing 1% NaCl (sodium chloride) (Merck-Millipore, U.S.A). A loopful of culture was dissolved in TE (Tris-EDTA) buffer (pH 8) (Sigma Aldrich, USA) followed by extracting DNA using phenol-chloroform-isoamyl alcohol mixture in the ratio 25:24:1 and alkaline pH and finally eluting DNA in nuclease-free water.

### Isolation of Genomic DNA From Stool Samples

Microbial genomic DNA was isolated from stool samples using QIAampUCP Pathogen Mini Kit (cat.no. 50214, Qiagen Inc., MD, USA), using the protocol provided by the manufacturer, for isolation of ultra-clean DNA. Accordingly, stool samples were subject to mechanical lysis using glass beads in a mini vortex-mixer (3020 Spinix, Tarsons) followed by enzymatic lysis using buffer containing pre-mixed bacterial cell-wall degrading enzyme cocktail under highly denaturing conditions at elevated temperatures of 70°C and protein removal and nuclease inactivation using a combination of Proteinase K (20 mg/ml) and buffer APL2 using spin columns (QIAamp UCP Mini Column, Qiagen Inc., MD, USA) wherein microbial nucleic acid is adsorbed on the silica membrane by centrifugation at 8,000 g for 1 min and washing with buffer containing ethanol followed by final elution using elution buffer containing Tris-EDTA (Ethylenediaminetetraacetic acid). The concentration of DNA from each sample was quantified using the NanoDrop Lite UV-Vis Spectrophotometer (Thermo Fisher Scientific, MA, USA) and diluted to obtain a uniform concentration of 25ng/ul for all samples using ultra-pure water obtained by purification with the Milli-Q® Integral Water Purification System (Merck-Millipore, USA) followed by autoclaving at 121°C for 15 min at 15psi. The DNA was used as template for PCR and Sanger sequencing. In the method described above salt and pH conditions help in the complete removal of proteins and other contaminants, which can inhibit downstream enzymatic reactions.

### AMR Profiling

AMR profile of each sample was obtained by PCR using specific primer set consisting of forward and reverse primers to amplify the gene of interest involved in antimicrobial resistance mechanism (Table 2). PCR was performed in a final reaction volume of 25 μl containing 1x GoTaq® Green master mix (cat.no. M7123, Promega Corporation, WI, USA), 0.4uMupstream and downstream primers and 1 ng/μl DNA template using a 96-well thermalcycler (GeneAmp® PCR system 9700, Applied Biosystems, Thermo Fisher Scientific, MA, USA). A list of genes and their corresponding primer sequences, annealing temperatures and size of the amplified fragment has been presented in Table 3. The PCR products were run on 1–1.5% agarose gel (depending on amplicon size) prepared using 1X TAE (Tris-acetic acid-EDTA) buffer followed by staining in ethidium bromide solution (10 mg/ml) and visualized in a UV-transilluminator (BioRad) and the results documented. For experimental control standard laboratory strains N16961, O395, and MO10 were used as template for the amplification of ARGs pre-documented as present or missing in these strains by previous reports archived in the public database NCBI. The amplicon size was determined using 100 bp and 1 kb DNA ladder (NEB, MA, USA and GeNetBio Corp., Korea).

**Table 2:** The eight major classes of antibiotics and Mobile Genetic Elements (MGEs) and their corresponding ARGs whose presence or mutations were determined in the study.

**ARG against class of antibiotic**	**ARG**
Tetracycline	*tetABCDEMG*
Macrolide	*ereAB, ermABC, mefABC, mphABCG, msrA*
Sulfamethoxazole	*sul2*
Aminoglycoside	*aadA1, aad2, aac(3), aac(6), strAB, aph*
Trimethoprim	*dhfr1, dfrA1, dfrA12, dfrA15, dfrA27, tmpC*
Amphenicol	*cat1, floR*
Carbapenemase	*ndm1,bla-_*VCC*1_*
Quinolones	*qnrVC, gyrA, gyrB, parC, parE*
MGEs	*Sxt-int, int1, int2, int4, tnpA, repA*

**Table 3:** Primers (5′-3′) used in the study.

**Primer**	**Gene/Description**	**Sequence**	**Annealing**	**Amplicon**	**References**
**Carbapenemase**					
*bla_*VCC*−1*F*_*	*bla-_*VCC*1_*	*ATCTCTACTTCAACAGCTCG*	55°C	755 bp	(2)
*bla_*VCC*−1*R*_*		*CCTAGCTGCTTTAGCAATCC*			
*ndm1-F*	*ndm1*	*CAATATTATGCACCCGGTG*	55°C	292 bp	(3)
*ndm1-R*		*GTGATTGCGGCGCGGCTAC*			
**Quinolones**					
*qnrVC-RD-F*	*qnr-VC*	*GGGGGCAAATTGCTTTGGTA*	60°C	220 bp	This study
*qnrVC-RD-R*		*TGAAGCGCCTCGAAGATTTG*			
**Aminoglycosides**					
*ant-F*	*ant(3″)-Ia /(add2)*	GCCTGAAGCCACACAGTGATA	51°C	660 bp	(4)
*ant-B*		CTACCTTGGTGATCTCGCCTT			
*aac(6′)-Ib-cr-Fw*	*6′-N-acetyltransferase*	*GGCGAATGCATCACAACTGG*	60°C	205 bp	This study
*aac(6′)-Ib-cr-Rev*		*AACCATGTACACGGCTGGAC*			
*aac(3)-FRD*	*aminoglycoside 3-N-*	*TGGCACTGTGATGGGATACG*	60°C	448 bp	This study
*aac(3)-RRD*	*acetyltransferase/aac(3)-I*	*CGTTTTCCAGGCGACTTCAC*			
*aadA1Fw-RD*	*Aminoglycoside*	*TTGGAAACTTCGGCTTCCCC*	60°C	101 bp	This study
*aadA1Rev-RD*	*adenylyltransferase/aadA1*	*TTAGCTGGATAACGCCACGG*			
*strB-F*	*strB*	*GGCACCCATAAGCGTACGCC*	60°C	470 bp	(5)
*strB-R*		*TGCCGAGCACGGCGACTACC*			
*strA-F*	*strA*	*TTGATGTGGTGTCCCGCAATGC*	60°C	383 bp	(5)
*strA-B*		*CCAATCGCAGATAGAAGGCAA*			
**Trimethoprim**					
*dfrA15-F*	*dfrA15*	*CAATGGGGGCTTTACCCAAC*	60°C	153 bp	This study
*dfrA15-R*		*CACCACCACCAGACACAATC*			
*dfrA12-F*	*dfrA12*	*CACGCTATCGCTTTGGCATC*	60°C	146 bp	This study
*dfrA12-F*		*ATTGGGAAGAAGGCGTCACC*			
*dhfrI F*	*dhfr1*	*CTGATATTCCATGGAGTGCCA*	57°C	434 bp	(4)
*dhfrI B*		*CGTTGCTGCCACTTGTTAACC*			
tmp-F	*dfr18*	*TGGGTAAGACACTCGTCATGGG*	60°C	389 bp	(6)
tmp-B		*ACTGCCGTTTTCGATAATGTGG*			
*dfr1-F*	*dfr18*	*CGAAGAATGGAGTTATCGGG*	60°C	372 bp	(7)
*dfr1-B*		*TGCTGGGGATTTCAGGAAAG*			
*dfrA27-F*	*dfrA27*	*TGGGGGCTCTCCCAAATAGA*	60°C	192 bp	This study
*dfrA27-R*		*TATGTAGCGTGTCGGCATGG*			
**Sulfamethoxazole**					
*sul2-F*	*sul2*	*AGGGGGCAGATGTGATCGAC*	60°C	625 bp	(6)
*sul2-B*		*TGTGCGGATGAAGTCAGCTCC*			
***Amphenicol***					
*floR-F*	*floR*	*TTATCTCCCTGTCGT TCCAGCG*	54°C	584 bp	(7)
*fLOR-2*		*CCTATGAGCACACGGGGAGC*			
*cat1-F*	*catI*	*GGCATTTCAGTCAGTTG*	55°C	627 bp	(5)
*cat1-B*		*CCGCCCTGCCACTCATC*			
**Macrolide**					
*msrA-F*	*msrA*	*GCACTTATTGGGGGTAATGG*	58°C	384 bp	(8)
*msrA-R*		*GTCTATAAGTGCTCTATCGTG*			
*mefA-F*	*mefA*	*AGTATCATTAATCACTAGTGC*	54°C	345 bp	(8)
*mefA-R*		*TTCTTCTGGTACTAAAAGTGG*			
*mefB-F*	*mefB*	*ATGAACAGAATAAAAAATTG*	45°C	1,255 bp	(9)
*mefB-R*		*AAATTATCATCAACCCGGTC*			
*mefC-F*	*mefC*	*ATGGAAAACCGTAAATGGTT*	55°C	885 bp	(9)
*mefC-R*		*TTAAATATTTTTGATTTTAC*			
*mphA-F*	*mphA*	*GTGAGGAGGAGCTTCGCGAG*	60°C	403 bp	(8)
*mphA-R*		*TGCCGCAGGACTCGGAGGTC*			
*mphB-F*	*mphB*	*GATATTAAACAAGTAATCAGAATAG*	58°C	494 bp	(8)
*mphB-R*		*GCTCTTACTGCATCCATACG*			
*mphG-F*	*mphG*	*ATGAAAAATAGAGATATTCA*	55°C	1,224 bp	(9)
*mphG-R*		*CTACTCAACACCTAACTGTA*			
*ermA-F*	*ermA*	*TCTAAAAAGCATGTAAAAGAAA*	52°C	533 bp	(8)
*ermA-R*		*CGATACTTTTTGTAGTCCTTC*			
*ermB-F*	*ermB*	*GAAAAAGTACTCAACCAAATA*	45°C	639 bp	(8)
*ermB-R*		*AATTTAAGTACCGTTACT*			
*ermC-F*	*ermC*	*TCAAAACATAATATAGATAAA*	45°C	642 bp	(8)
*ermC-R*		*GCTAATATTGTTTAAATCGTCAAT*			
*ereA-F*	*ereA*	*GCCGGTGCTCATGAACTTGAG*	60°C	420 bp	(8)
*ereA-R*		*CGACTCTATTCGATCAGAGGC*			
*ereB-F*	*ereB*	*TTGGAGATACCCAGATTGTAG*	55°C	537 bp	(8)
*ereB-R*		*GAGCCATAGCTTCAACGC*			
Aph F	*aphA*	*GGCAATCAGGTGCGACAAT*	52°C	484 bp	(5)
Aph R		*GTGACGACTGAATCCGGTGA*			
**Tetracyclines**					
*tetA-F*	*tetA*	*GTAATTCTGAGCACTGTCGC*	62°C	957 bp	(10)
*tetA-R*		*CTGCCTGGACAACATTGCTT*			
*tetB-F*	*tetB*	*CTCAGTATTCCAAGCCTTTG*	57°C	436 bp	(10)
*tetB-R*		*CTAAGCACTTGTCTCCTGT*			
*tetC-F*	*tetC*	*TCTAACAATGCGCTCATCGT*	62°C	589 bp	(10)
*tetC-R*		*GGTTGAAGGCTCTCAAGGGC*			
*tetD-F*	*tetD*	*ATTACACTGCTGGACGCGAT*	57°C	1,124 bp	(10)
*tetD-R*		*CTGATCAGCAGACAGATTGC*			
*tetE-F*	*tetE*	*GTGATGATGGCACTGGTCAT*	62°C	1,199 bp	(10)
*tetE-R*		*CTCTGCTGTACATCGCTCTT*			
*tetG-F*	*tetG*	*TTTCGGATTCTTACGGTC*	55°C	856 bp	(11)
*tetG-R*		*TCCTGCGATAGAGCTTAGA*			
*tetM-F*	*tetM*	*GTRAYGAACTTTACCGAATC*	55°C	634 bp	(11)
*tetM-R*		*ATCGYAGAAGCGGRTCACT*			
**MGEs**					
*sxt-F*	SXT Integrase	*TTATCGTTTCGATGGC*	50°C	800 bp	(10)
*sxt-B*		*GCTCTTCTTGTCCGTTC*			
*int-1U*	Integron class1	*GTTCGGTCAAGGTTCTG*	50°C	923 bp	(5)
*int-1D*		*GCCAACTTTCAGCACATG*			
*int-2U*	Integron class 2	*ATGTCTAACAGTCCATTTT*	50°C	450 bp	(5)
*int-2D*		*AAATCTTTAACCCGCAAAC*			
*int-4U*	Integron class 4	*GTGTTCGCGAATTTATGC*	50°C	936 bp	(5)
*int-4D*		*ACGGGATAATGGGCTTAA*			
*tnpA-F*	*tnpA*	*GAATCTCAGCAGGCAATGCG*	55°C	545 bp	This study
*tnpA-R*		*GCCAAYTTGCCAGACTGGTG*			
*repA-F*	*repA*	*CGTTGGGGTTCATCAATG*	55°C	1,000 bp	(12)
*repA-R*		*GACTCACCGCAAATGAGC*			
**Sequencing**					
*130*	16S rDNA C1-C9	*GGCGGATCCAAGGAGGTGTTCCAGCCGC*	55°C	1,500 bp	(13)
*139*		*GGCCTCGAGAGAGTTTGATCCTGGCTCAGG*			
*S-D-Bact-0341-b-S-17*	16S rDNA V3-V4	*CCTACGGGNGGCWGCAG*	55°C	464 bp	(14)
*S-D-Bact-0785-a-A-21*		*GACTACHVGGGTATCTAATCC*			
*gyrA_seqFOR2*	*gyrA*	*TCTTCCTGATGTGCGTGATG*	55°C	771 bp	This study
*gyrA_seqREV2*		*GCACTGATCCCTTCGACTTT*			
*gyrB_seqFOR*	*gyrB*	*TGAAAGTGCCGGATCCTAAAT*	55°C	662 bp	This study
*gyrB_seqREV*		*GTACTGCTCTTGTTTGCCTTTC*			
*parC_seqFOR*	*parC*	*CGCAAATTTACTGAAGACGCTTATC*	55°C	729 bp	This study
*parC_seqREV*		*CACGATATCCGAGCCTTCTTTG*			
*parE_seqFOR*	*parE*	TGCTGGCCAAACCAAAGA	55°C	640 bp	This study
*parE_seqREV*		CTGATCATCGAGTGCGTAGAAC			

### Sanger Sequencing for Confirmation of ARGs

PCR products obtained above and containing the amplicon of interest was purified using the Wizard® SV Gel and PCR Clean-Up System (cat. no. A9281, Promega Corporation, WI, USA) that can purify100 bp to 10 kb DNA fragments with Wizard® SV Minicolumns. DNA quantity and quality was adjusted to fulfill the requirements for Sanger sequencing. Purified PCR products with optimum absorbance ratios (A_260/280_) between 1.8 and 2.0 were selected to serve as template. Cycle sequencing was carried out with the BigDye^TM^ Terminator v3.1 cycle sequencing kit (cat. no. 4337455, Applied Biosystems, Thermo Fisher Scientific, MA, USA) using 10 μl reaction mixture containing BigDye™Terminator v3.1 Ready Reaction Mix, BigDye™Terminator v3.1 Sequencing Buffer, final concentration of 3.2 pMol of either forward or reverse primer and DNA template quantity of 1–5 ng depending on the size of the fragment being sequenced. Cycling conditions consisted of denaturation at 96°C for 1 min and 25 cycles of denaturation, annealing, and extension at 96°C for 10 s, 50°C for 5 s, and 60°C for 4 min, respectively. The cycle sequencing product was purified using 3M sodium acetate solution (pH 4.8) and absolute ethanol (Emsure, Merck, Germany) and washed with 70% ethanol and finally vacuum dried. The dried pellet was resuspended in 10 μl Hi-Di™ Formamide and loaded onto the Applied Biosystems™ 48-capillary DNA Analyzer 3730 (Thermo Fisher Scientific, MA, USA) for sequencing and collection of data using the recommended signal to noise ratio. Accordingly, unambiguous clear curves with sharp peaks indicating clear base-calling and low background noise were considered. Curves with minimum signal 300 for all the bases and maximum noise 20 were set as the threshold for accepting raw data. Chromatograms considered for analysis had maximum signal in the range of *G* = 964–4,176; *A* = 518–2,967; *T* = 537–2,435; *C* = 662–4,186. Noise was confined to baseline (signals 0–50) and did not interfere with data interpretation. Raw data was viewed and analyzed using SeqMan™II (version Windows 32 SeqMan 5.01) software (DNASTAR Inc., WI, USA) and assembled sequence data in the clear range was accepted and considered. This was subject to local alignment using Megablast program of Nucleotide BLAST (Basic Local Alignment Search Tool) on the NCBI (National Center for Biotechnology Information) server.

### Calculation of Relative Abundance and Frequency of ARGs

Relative abundance of ARGS present in fecal samples was calculated. For each ARG it was represented as the percentage of fecal samples found to be carrying that particular ARG. This can be represented by the formula:

For *ARG A*

(Number of fecal samples positive by PCR for ARG A/ n)^*^100

Where ARG A is any ARG which has been included in the study and PCR positive and n is the total number of fecal samples used in the study. Here, n is 25.

Frequency of occurrence of resistance marker was calculated by the formula:

For any particular class of antibiotic:

(Number of fecal isolates found positive for at least one ARG marker against that class / n)^*^ 100

Where, n is the total number of fecal samples used in the study. Here, n is 25.

On the basis of these two parameters described above we concluded which group of antibiotics has the highest abundance of resistance marker.

### 16S Conserved and Variable Region Amplification

PCR amplification for confirming the presence of bacterial DNA in the DNA isolated from fecal specimens was carried out for the conserved regions C1-C9 and variable regions V3-V4 of 16S rDNA using standard primer sets targeting these regions. PCR amplification was carried out in 25 μl final reaction volume using the same reagents described above. The cycling conditions for PCR using primer sets 130–139 and S-D-Bact-0341-b-S-17-S-D-Bact-0785-a-A-21 were described by Bag et al. (13) and Klindworth et al. (14), respectively.

### Sanger Sequencing of 16s rDNA Variable Region

For representative samples the 464 bp fragment obtained by PCR amplification of variable region V3-V4 of 16S rDNA with primer set S-D-Bact-0341-b-S-17- S-D-Bact-0785-a-A-21 was subject to Sanger sequencing. The PCR product was purified and subject to cycle sequencing reaction and finally sequenced using the 48-capillary DNA Analyzer 3730 (Thermo Fisher Scientific, MA, USA) following the same procedure described above. Sequence assembly and analysis was done using SeqManII software (DNASTAR Inc., WI, USA) and the assembled sequence data in the clear range was accepted and considered. With the help of the Megablast program of the local alignment tool Nucleotide BLAST on the NCBI server the sequenced data was matched with archived and annotated 16S rDNA sequences available in GenBank, the NIH genetic sequence database.

### Routine Isolation, Laboratory Culture, and Antibiotic Susceptibility Test of Diarrheal Pathogens

The enteric pathogen associated with diarrheal etiology in each fecal sample was isolated using enrichment technique. Accordingly, the samples were streaked onto selective and differential media plates for the detection of common diarrheal pathogens, *Vibrio* sp*., E. coli, Salmonella* sp., *Shigella* sp., *Aeromonas* sp., *Campylobacter* sp. in the Bacteriology Division Laboratory at NICED. Accordingly, bacterial culture plates TCBS, HEA (Hektoen enteric agar), XLD (Xylose Lysine Deoxycholate), MacConkey, Blood agar were used for each fecal specimen. Culture plates were incubated overnight at 37°C (3–5 days for *Campylobacter* sp.) and colonies from the plates were subject to biochemical test using Triple Sugar Iron Agar for identification of acid (lactose/sucrose fermenting and non-fermenting), gas, and hydrogen sulfide producing organisms. Single colonies from TCBS plates were subjected to oxidase and string test for the confirmation of *V. cholerae*.

Confirmed *V. cholerae* strains were further tested for antibiotic susceptibility by Kirby-Bauer Disc Diffusion method. The strains were grown on LA (Luria agar) plates overnight at 37°C and a loopful of culture was taken from the plate and inoculated into Luria broth and OD of this culture suspension was adjusted to obtain OD of 0.5 McFarland. With the help of autoclaved swab stick bacterial culture from this broth was spread onto Mueller-Hinton Agar (MHA) plates and allowed to dry in a biosafety cabinet. Antibiotic discs (BD BBL™, NJ, U.S.A.) were placed on the surface of MHA plates with sterile forceps to test sensitivity toward common antibiotics, namely, ampicillin (10 μg), ceftriaxone (30 μg) chloramphenicol (30 μg), ciprofloxacin (5 μg), gentamicin (30 μg), ofloxacin (5 μg), imipenem (10 μg), azithromycin (15 μg), gentamicin (10 μg), norfloxacin (10 μg), nalidixic acid (30 μg), streptomycin (10 μg), SXT (trimethoprim-sulfamethoxazole) (1.25, 23.75 μg), and tetracycline (30 μg). The plates were incubated overnight at 37°C. The diameter of the clear zone (zone of inhibition) around each disc was measured, recorded and compared with the zone diameter interpretive chart provided by the manufacturer and which follows CLSI (Clinical and Laboratory Standards Institute) guidelines^2^. Accordingly, the strains were categorized as resistant, intermediate or susceptible.

### Sanger Sequencing for Detecting Single Nucleotide Polymorphisms (SNPs) in Genes Involved in Quinolone Sensitivity

Four primer sets *gyrA_seqFOR2-Rev2 gyrB_seqFOR-Rev, parC_seqFOR-Rev, parE_seqFOR-Rev* (Table 2) were designed to amplify regions of genes encoding Topoisomerase II subunits A and B (*gyr A* and *gyrB*) and Topoisomerase IV subunits A and B (*parC* and *parE*). Sequencing was carried out to study SNPs in quinolone resistance determining regions (QRDR) in fecal DNA from representative samples. Amplification of fragments of *gyrA, gyrB, parC*, and *parE* was carried out by PCR with primers *gyrA_seqFOR2-Rev2, gyrB_seqFOR-Rev, parC_seqFOR-Rev, parE_seqFOR-Rev* which were designed from the N16961 sequence (reference sequence ID. NC_002505; Gene ID: 2615137) to amplify 771 bp fragment of *gyrA*, 662 bp fragment of *gyrB*, 729 bp fragment of *parC* and 640 bp fragment of *pare* which were purified using the Wizard® SV Gel and PCR Clean-Up System (cat. no. A9281, Promega Corporation, WI, USA) and used as template for cycle sequencing reaction with the BigDye^TM^ Terminator v3.1 cycle sequencing kit (cat. no. 4337455, Applied Biosystems, ThermoFisher Scientific, MA, USA) and were sequenced on the Applied Biosystems™ 48-capillary DNA Analyzer 3730 (Thermo Fisher Scientific, MA, USA) in the same manner as already described in the preceding section (Sanger Sequencing for confirmation of ARGs). Sequence data was assembled with SeqMan II software (DNASTAR Inc., WI, USA) and the sequence identity was matched by global alignment with the corresponding genomic regions of standard *V. cholerae* O1 El Tor strain N16961 to map single nucleotide polymorphisms (SNPs).

## Result

### 16S Conserved Region PCR

A 1,500 bp amplicon of conserved region of 16S rDNA was obtained spanning the C1-C9 region of bacterial genomic DNA in fecal samples on PCR amplification with primer set *130–139*. The results confirmed the presence of bacterial DNA (Figure S1).

### AMR Profile

PCR was performed targeting a group of 49 genes associated with 8 different classes of common antibiotics like carbapenem, tetracycline, quinolones, aminoglycosides, macrolide, trimethoprim, sulfamethoxazole, amphenicol, and components of mobilome like transposase, SXT integrase, and markers of classes 1, 2, and 4 integron. PCR results showed amplification of genes *aadA1, aad2, strAB, aac* *(**2**), aac(6*′*)-Ib-cr, tetABCDEM, SXT integrase, floR, dhfr1, dfrA1, dfrA12, dfrA15, cat1, sul2, aph, ereA, mefA, mphA, tnpA, int1, int2, int4*. This revealed that the microbial genomic DNA bore ARG markers against aminoglycoside, tetracycline, macrolide, chloramphenicol, trimethoprim, sulfamethoxazole, and mobile genetic elements (MGEs). Table 1 shows the results obtained on screening for ARGs and the different classes of antibiotics against which they are active. All of the 25 samples tested for the presence of ARGs, showed genetic determinants associated with resistance against at least two classes of these broad-spectrum antibiotics. Where standard control strains were not available to compare and confirm the results, PCR products were sequenced using Sanger Sequencing to determine and confirm the identity of the gene. Accordingly, genes *tetABCDEM, aph, dhfr1, mphA, mefA, dfrA12, tnpA, aadA1, aad2, aac(3)/(aacC), aac(6*′*)-Ib-cr, ereA, int1, int2* were sequenced and the assembled sequence on local alignment using Megablast program of Nucleotide BLAST confirmed the identity of these genes thereby confirming their presence in the fecal samples of diarrheal patients in Kolkata and suburban areas. Representative gel pictures showing the results of ARG profiling are available in Figures S2–S4. The assembled sequences and the results obtained on alignment of these sequences using BLAST are available as Supplementary Material S5.

**Figure 1:**
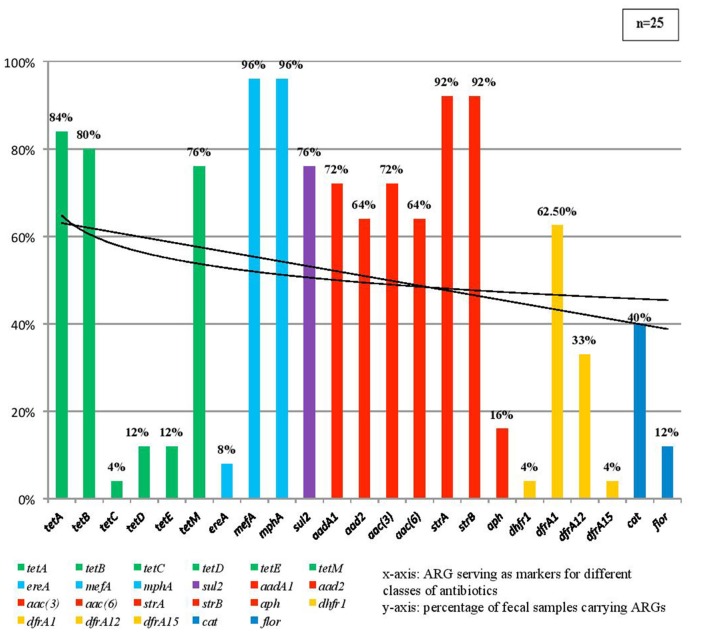
This figure showing the percentage of fecal samples found positive by PCR for each individual antimicrobial resistance marker.

**Figure 2:**
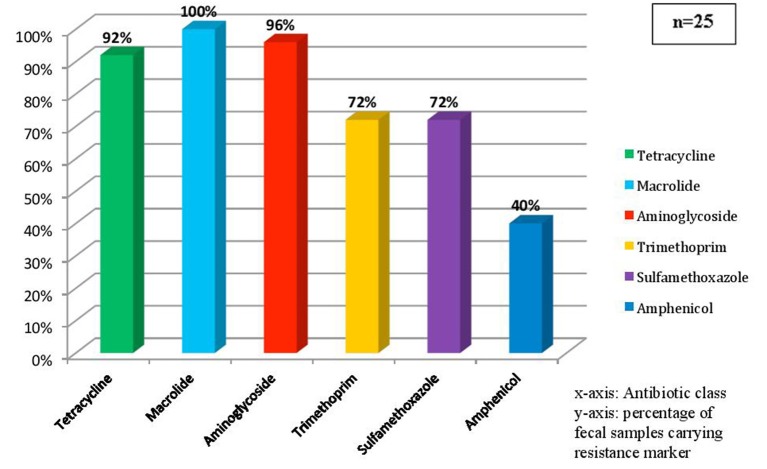
This figure showing the percentage of fecal sample carrying resistance determinants against each class of antibiotics.

The relative abundance of different ARGs (Figure 1) and the frequency of occurrence of resistance marker against each class of antibiotic (Figure 2) were calculated. It was found that 96% samples carried *mphA* and *mefA* genes encoding resistance against macrolide and hence these two genes were found to have highest occurrence followed by 92% occurrence of *strA* and *strB* encoding aminoglycoside resistance followed by tetracycline resistance due to the occurrence of 84% isolates with *tetA* gene, 80% isolates with *tetB* gene and 76% isolates with *tetE* gene. Other genes with high occurrence rate are *cat1* for chloramphenicol resistance (40%), *dfrA1* (62.5%), *dfrA12* (33%) both encoding trimethoprim resistance, *aad2* (64%), *aadA1* (72%), *aac(3)* (72%), *aac6*(64%) all encoding resistance against aminoglycosides. Lower rate of occurrence was seen among *tetC* (4%), *ereA* (8%), *tetD* and *tetE* (12% each), *floR* (12%), *dhfr1* and *dfrA15* (4% each), and *aph* (16%).

In addition 3 isolates showed the presence of SXT integrase, 19 isolates showed the presence of *tnpA* encoding transposase and 21 isolates carried integron class1, 11 isolates carried integron class 2 and 8 isolates carried integron class 4 and 4 isolates were found to carry all the three classes of integrons showing the presence of MGEs and signatures associated with MGEs.

### Isolation of Diarrheal Pathogens by Conventional Culture and Antibiotic Sensitivity Test of *V. cholerae*

Pathogens responsible for causing diarrhea in the patients were isolated from the fecal samples by conventional culture method and confirmed using routine biochemical tests. Table 1 shows pathogens isolated from each fecal sample. *V. cholerae* O1 strains were subject to antibiotic sensitivity test by Kirby-Bauer Disc Diffusion method. The antibiogram results were compared with results of PCR AMR profile of corresponding fecal sample from which the strain was isolated (Table 4). On comparison of ARG profile of feces with the antibiogram of its corresponding pathogen it was clear that fecal samples carried ARGs which were not always being expressed by pathogens. At the same time even the pathogen's antibiogram revealed resistance against those classes of antibiotics against which resistance markers were not detected by PCR. Fecal sample KOL18B3-1 carried the following genes *tetABM, cat1, floR, ant, dfrA12, dfr1, aac(3), aac6*′*, aadA1, strAB, mphA, mefA, tnpA, intI1, intI2, intI4, sxt* but the antibiogram of the *V. cholerae* strain isolated from this fecal sample showed that the strain was not resistant against tetracycline and macrolides. Similarly, *V. cholerae* strains isolated from fecal samples KOL18B2-1 and KOL18B2-4 were sensitive to chloramphenicol but the samples showed the presence of gene *cat1*, which affords resistance against chloramphenicol.

**Table 4:** Comparison of ARG profile ofilef cholera stool and antibiogram of *V. cholerae* isolated from it.

**Stool sample**	**Pathogen isolated**	**AMR profile of fecal isolate by ARG profile**	**Resistance profile by antibiogram of pathogen**
KOL18B2-1	*VC O1 Inaba*	*tetAB, cat 1, sul2, ant, dfrA12, aac(3), aac6′, aadA1, strAB, mefA, mphA, tnpA, int1, int4*	Tetracycline, SXT, nalidixic acid, streptomycin
KOL18B2-2	*VC O1 Ogama, C. jejuni*	*tetABM, cat1, floR, sul2, dhfr1, ant, dfrA12, dfr1, aac(3), aac6′, aadA1, strAB, mphA, tnpA, int1, int2, int4, sxt*	*SXT, nalidixic acid, streptomycin, chloramphenicol
KOL18B2-4	*VC O1 Inaba, Campylobacter sp*.	*tetABM, cat1, sul2, ant, dfrA12, aac(3), aac6′, aadA1, strAB, aph, mefA, mphA, tnpA, int1, int4*	*Ampicillin, tetracycline, SXT, nalidixic acid, streptomycin
KOL18B3-1	*VC O1 Ogama*	*tetABM, cat1, floR, ant, dfrA12, dfr1, aac(3), aac6′, aadA1, strAB, mphA, mefA, tnpA, int1, int2, int4, sxt*	SXT, nalidixic acid, streptomycin, chloramphenicol

VC, V. cholera. Antibiogram for VC*.

KOL18B2-6, KOL18B2-7, KOL18B3-2, KOL18B3-4, KOL18B3-7, KOL18B3-9, KOL18B3-10, KOL18B3-16, KOL18B3-17 failed to produce conclusive results by culture method for the identification of any pathogen that we routinely test for diagnosis. However, from all of these samples, resistance determinants were detected successfully by PCR indicating the presence of bacterial DNA and ARGs (Table 5).

**Table 5:** Fecal samples with resistome but no culturable pathogen.

**Stool sample**	**Pathogen isolated**	**ARG profile**
KOL18B2-6	No pathogen	*tetABM,sul2,aadA1,strAB,mefA,mphA*
KOL18B2-7	No pathogen(MAC LF)	*tetABDM,sul2,dfr1,ant,aac(3),aac(6′),aadA1,strAB,mefA,mphA,tnpA,int1,int2*
KOL18B3-2	*No pathogen*	*tetABM,cat1,ant,dfrA12,aac(6′),aadA1,strAB,mphA,mefA,int1,int4*
KOL18B3-4	*HEA (Green)TSI(k/A+G)*	*tetAB,cat1,sul2,ant,dfr1,aac(3),aac(6′),aadA1,strAB,mefA,mphA,tnpA,int1,int2*
KOL18B3-7	No pathogen(MAC LF)	*tetABM,dfr1,aac(3),strAB,mefA,mphA,tnpA,int2*
KOL18B3-9	*HEA (Green)TSI(k/A+G)*	*tetABM,sul2,ant,dfrA12,aac(3),aac(6′),aadA1,strAB,mefA,mphA,tnpA*
KOL18B3-10	*Mac (NLF)TSI(k/A+G)*	*tetABM,sul2,strAB,mefA,mphA,tnpA*
KOL18B3-16	No pathogen(MAC LF/NLF)	*tetA,sul2,aac(3),aac(6′),strAB,mefA,mphA,tnpA*,
KOL18B3-17	No pathogen(MAC LF/NLF)	*tetABM,sul2,aac(3),aac(6′),strAB,mefA,mphA,tnpA*,

### 16S Variable Region Sequencing

Four hundred sixty-four bp amplicon from the V3-V4 region of 16S rDNA of representative samples was obtained by PCR and subject to Sanger sequencing. Accordingly DNA from nine samples namely, KOL18B2-1, KOL18B2-2, KOL18B3-1, KOL18B3-2, KOL18B3-3, KOL18B3-9, KOL18B3-10, KOL18B3-11, KOL18B3-12 and genomic DNA from two laboratory strains of *V. cholerae* namely O395 and N16961 were used for the amplification of 464 bp amplicon which was subject to nucleotide sequencing on the ABI platform and the sequence data was subject to pairwise alignment using local alignment software Nucleotide BLAST on the NCBI server. From KOL18B3-2, KOL18B3-3, and KOL18B3-10 no pathogen had been isolated by culture but they carried ARGs as revealed by PCR. The assembled sequence data obtained from sequencing of DNA from all of the above fecal samples could be successfully aligned with known annotated 16S ribosomal DNA sequences archived in the GenBank public database (Table 6). The results revealed significant information by indicating the presence of phyla *Bacteroidetes, Firmicutes*, and *Proteobacteria* (classes β*-proteobacteria* and γ*-proteobacteria)* in the fecal samples from Kolkata. The 16S variable region DNA sequencing revealed that a majority of fecal samples carried DNA from family *Enterobacteriaceae* as 16S rDNAamplicons from KOL18B2-1, KOL18B3-9, and KOL18B3-12 aligned 92–100% with 16S rDNA of *Eschericia* sp. and KOL18B3-2, matched 99% to uncultured *Eschericia* sp. KOL18B2-2 contained DNA that matched 89% with *Bacteroidetes* DNA. KOL18B3-3 and KOL18B3-10 matched up to 98% with DNA from *Bifidobacteriaceae* and KOL18B3-11 carried DNA from *Streptococcus* sp. revealing the presence of *Firmicutes* in KOL18B3-3, KOL18B3-10, and KOL18B3-11. KOL18B3-1 carried DNA that matched with uncultured β*-proteobacterium* and uncultured *Firmicutes* (*Clostridium* sp.). 5 out of 9 representatives and therefore >55% carried DNA from phylum *Proteobacteria*, 4 out of 9 samples and hence >44% carried DNA from phylum Firmicutes and 1 out of 9 fecal DNA samples and hence 11% revealed the presence of DNA from phylum Bacteroidetes. These results are available in Figure S6. The assembled sequences from O395 and N16961 used as experimental control matched 100% with *V. cholerae* 16S rDNA. The assembled sequences are available as Supplementary Material S7.

**Table 6:** 16S rDNA V3-V4 region sequence identity of OTU obtained using Megablast.

**Stool Sample**	**Pathogen isolated**	**16S rDNA V3-V4 identity (BLAST)**
KOL18B2-1	*VC O1 Inaba*	100% to *Eschericia* sp.
KOL18B2-2	*VC O1 Ogawa+C.jejuni*	89% to uncultured bacterium and uncultured Bacteroidetes
KOL18B3-1	*VC O1 Ogawa*	88-90% to uncultured β-proteobacterium, uncultured *Clostridium* sp.
KOL18B3-2	*N pathogen*	99% to uncultured *Eschericia* sp.
KOL18B3-3	*EAEC*	98% to *B. breve, B. longum, B. fecale, B. adolescentis*, uncultured *Bifidobacterium*
KOL8B3-9	*HEA (Green)TSI(k/A+G)*	92% to *E. coli*, uncultured *Enterobacteriaceae*
KOL18B3-1	*Mac (NLF)TSI(k/A+G)*	88% to uncultured *Bifidobacteriaceae*and 90% to uncultutred bacterium clone
KOL18B3-11	*Aeromonas* sp.	93% to *Streptococcus thermophilus*, uncultured *Streptococcus* sp., *uncultured organism*
KOL18B3-12	*Aeromonas* sp.	99% to *Escherichia coli*

### Mutations in Quinolone Resistance Determining Regions (QRDR) of Topoisomerase II and IV Genes

Quinolone resistance develops due to mutations in the genes encoding topoisomerase II and IV. Twelve samples were subject to SNP analysis. From these fecal samples different diarrheal pathogens had been isolated by culture method. Accordingly, KOL18B2-1, KOL18B2-2, KOL18B2-4, KOL18B2-5, KOL18B3-1, KOL18B3-2, KOL18B3-3, KOL18B3-5, KOL18B3-12, KOL18B3-13, KOL18B3-14, and KOL18B3-15 were included for studying SNPs in the QRDRs. Clear amplification results showing presence of Topoisomerase II and IV genes of *V. cholerae* origin was obtained (for gel picture refer to Figure S8) by PCR using primers *gyrA_seqFOR2-Rev2, gyrB_seqFOR-Rev, parC_seqFOR-Rev, parE_seqFOR-Rev* which were designed from the N16961 sequence (reference sequence ID: NC_002505.1) Samples used were KOL18B2-1 for *gyrB*, KOL18B2-2, and KOL18B3-1 for *gyrAB, parCE*. These were the samples from which *V. cholerae* was isolated as the diarrheal pathogen. Amplified fragments of *gyrAB, parCE* from KOL18B2-2 and KOL18B3-1 were subject to SNP analysis by Sanger sequencing. Accordingly 771 bp fragment of *gyrA*, 662 bp fragment of *gyrB*, 729 bp fragment of *parC* and 640 bp fragment of *parE* were sequenced on the ABI platform. The assembled data obtained using the SeqMan II software was subject to pairwise alignment with the nucleotide sequence of the corresponding regions in the N16961 genome available in GenBank on NCBI (NC_002505.1) The result of pairwise alignment revealed 100% pairwise match of nucleotide sequence between the assembled 586 bp *gyrA* fragment of KOL18B3-1 with that of N16961 (NC_002505.1) corresponding to positions 1754436 to 1755021 at the locus VCO1597. The assembled fragment of *gyrB* subunit from KOL18B2-2 was of length 626 bp and it showed 99.84% identity to the corresponding 626 bp region of gene *gyrB* at the locus VC00005 positions 4668–5295 of the reference sequence *V. cholerae* strain N16961 (NC_002505.1) chromosome 1 (accession no. CP028827.1). The alignment presented a gap showing a deletion at the 16th position of the aligned fragment which corresponded to position 5280 from the 3′ end in the reference sequence that has an “A.” The assembled and edited fragments of *parC* and *parE* from the same sample were of length 322 and 594 bp. These showed 100% identity to 322 bp fragment of *parC* gene at locus VC00463 and to 594 bp fragment of *parE* gene at locus VC00462 in the N16961 sequence (accession no. CP028827.1) on pairwise alignment using BLAST. The assembled and edited 420 bp fragment of *pare* gene from KOL18B3-1 was aligned with reference sequence N16961 (accession no. CP028827.1) and also with *parE* sequence from KOL18B2-2. The pairwise alignment showed this region corresponded to positions 476083–476502 in the reference sequence. This 420 bp fragment of *parE* from KOL18B3-1 showed 99.76% sequence identity to both. A single base substitution was observed at position corresponding to position 476151 of reference sequence where in “*A*” in the reference sequence had been replaced by “*T*” in the fecal DNA sequence. The same difference was noticed in KOL18B3-1 when its nucleotide sequence of the same genomic region of 420 bp was compared with that of KOL18B2-2 that lacked this SNP. The nucleotide change was not observed in KOL18B2-2and it bore 100% identity to reference sequence over this genomic region.

## Discussion

AMR due to MDR has emerged as a critical obstacle to the treatment of enteric infections like diarrhea. Therefore, research directed toward leveraging this problem is an urgent need of the hour. The study described here has provided crucial insights into the gut microbiota composition and its resistome to understand transmission of ARGs between pathogens and microbiota. For a complete understanding of the microbiome, metagenomic analysis by next-generation sequencing (NGS) is the key that enables intensive analysis. However, it is an expensive technique and may not be within the affordability and outreach of a majority of laboratories, particularly in developing countries. In such situations, common molecular tools like PCR and Sanger sequencing, used decisively can produce far fetching outcome.

We have used these simple yet useful and robust methods to study the ARG composition in diarrheal fecal samples and to identify major members of the intestinal microbiota of diarrheal patients from Kolkata and the suburban areas. The QIAamp UCP Pathogen Mini Kit is a high quality kit for microbial DNA extraction and purification from small volumes of body fluids in only two hours. This kit provides high yield of DNA, requires no downstream purification and is compatible with Sanger Sequencing and deep sequencing techniques. In addition, since this kit is used for microbial DNA extraction from body fluids and urine, it is perfectly suitable for diarrheal stool which is liquid too. Fecal samples contain DNA from different members of the fecal microbiota and in such a polymicrobial community it is most likely that the most abundant member will be identified on the basis of Sanger sequencing of its 16S ribosomal DNA marker. This technique has been used for more than two decades for identification of bacterial taxa and also of unculturable bacteria for phylogenetic study (15–17). However, this is also one limitation of phylogenetic analysis by Sanger Sequencing of 16S rDNA. Since one of the major goals of the study was to identify the most abundant phylum present in the samples and to detect the presence of bacterial DNA in samples which were found to be devoid of any culturable bacterial pathogen which we tested using pure culture technique 16S rDNA Sanger Sequencing method was the method of choice as an alternative to NGS and was applied to achieve our goal. The variety of taxonomic entities identified in a sample depends largely on the primers selected for amplification of 16S rDNA. The efficiency of different primers has been scrutinized by several groups who have attempted to select the one with the best coverage of bacterial taxa (14–16). The primers we used in the study to examine OTUs have been reported to be the most suitable for bacterial identification up to the group and genus levels and in identifying the most abundant groups (14) thereby serving our purpose of identification of the most abundant phyla. Diarrhea like other enteric infectious diseases is accompanied with dysbiosis of the microbiome. Our study indicates that in majority of diarrheal patients proteobacteria is the dominant phylum followed by firmicutes suggesting a dysbiotic microbiota with proteobacterial “blooms” which is a characteristic of an abnormal microbiota (18). Detection of a spectrum of 49 ARGs was carried out to study resistance against major classes of antibiotics recommended for the treatment of diarrhea. Primers used for the study were selected or designed from whole genome sequences of different organisms archived in GenBank, NCBI for different classes of antibiotics based on information provided by previous published reports of other workers indicating the source or origin of these genes. ARGs involved in resistance against tetracyclines, macrolides, and aminoglycosides were found to be the major components of the resistome irrespective of the diarrheal pathogen isolated or the major operational taxonomic unit (OTU) present in any particular fecal isolate. The ARGs *mphA* and *mefA* encode phosphotransferase and efflux pump, respectively. The phosphotransferase is involved as a modifying enzyme which inactivates macrolides. These genes are active against erythromycin and azithromycin (8). Both these genes have been found to be abundantly present in gram negative bacteria according to published reports by other groups (8). Primers used to amplify these genes were designed from *Enterobactericeae* nucleotide sequences (8). These genes have been reported to be plasmid-borne and have been found to be transferred from *E. coli* to *Shigella* sp. (8). In our study we found from the antibiogram of *V. cholerae* strains that these were sensitive toward macrolides. These were isolated from samples KOL18B2-1, KOL18B2-2, KOL18B2-4, KOL18B3-1. However, in these samples these genes were found to be present. The study helped us to predict the future possibility that these ARGs present in the gene pool may at any point of time be transferred into organisms like *Vibrio cholerae* which come in contact with them and confer resistance upon these pathogens and contribute to the spread of resistance of macrolide drugs which are recommended last resort drugs for treatment of diarrheal diseases like cholera. *mefC* and *mphG* which were examined using primers designed from genomic sequence of *Photobacterium damselae* subsp*. damselae* strain 04Ya311 (accession no. AB571865) (9), representing ARGs from marine bacteria, were not detected in any fecal sample. Our results demonstrated that among tetracycline resistance determinants classes A and B were widespread among the clinical samples, which were examined. KOL18B2-3, KOL18B2-7, and KOL18B3-12 were found to carry class D tetracycline resistance gene and KOL18B2-3, KOL18B3-3, and KOL18B3-12 were found to carry class E tetracycline resistance gene. Classes D and E tetracycline resistance gene have been reported to be widespread among environmental gram-negative bacteria from marine sediment and fish intestine (19, 20) and are not often isolated from clinical strains. Tetracycline resistance gene was first isolated from *Shigella dysenteriae* (21). Latter studies showed they were widespread among families of class γ-Proteobacteria like *Enterobacteriaceae, Vibrionaceae, Aeromonadaceae, and Moraxellaceae*. They have been reported in clinical and environmental isolates of these classes and have been isolated from *Acinetobacter* sp., *Vibrio* sp., and *Aeromonas sp*. in addition to *E. coli* and *Salmonella* sp. (11, 22). The tetracycline resistance determinants are found on MGEs like plasmids and transposons and have been found to be effectively transferred among bacterial species by conjugation. The resistance determinants selected for this study are involved in efflux of tetracyclines except class M which may have a function in protection of bacterial ribosomes against tetracycline action. Class M appeared in a number of samples used in this study and has been previously reported in a number of gram-positive and gram-negative genera (21). The primers used in this study for the detection of all these genes were designed from members of family *Enterobacteriaceae* like *E. coli* and *S. Ordonez* and were of plasmid or transposon origin (11).

The human intestine is an optimum environment for conjugal transfer of genetic components among bacterial members. ARGs present in feces and representing the intestinal resistome may be easily transferred to pathogen genome and contribute to AMR development against those drugs in the future. From the presence of components of mobilome (SXT integrase, three different classes of integrons and transposase) one can forebode that ARGs present in the fecal microbiome are prone to dissemination by HGT, which is a common phenomenon in bacteria.

In the context of cholera, which is a common diarrheal illness in poverty-stricken parts of the world, predominantly in the low and middle income countries, the presence of a gene pool rich in ARGs in cholera stool against the most potential classes of antimicrobials namely, macrolides, tetracyclines, and aminoglycosides which are the drugs of choice in the treatment of cholera is highly alarming. The strains of *V. cholerae* used for antibiogram analysis were found to be sensitive to these drugs. However, the presence of resistance markers against these antimicrobials in the corresponding fecal resistome and in the population on the whole can admonish of grave danger lurking in the near future when these ARGs can well find their way into the genome of *V. cholerae* and contribute to resistance. HGT is a common occurrence in *V. cholerae*, which occurs when the organism passes through the human gut and also in the environment (23). It is a bacteria with dual lifestyle and can be an important agent in the transmission of ARGs between the gut and the environment (24).

Another significant finding of our study is the coexistence of 16S rDNA signature of *Bifidobacteria* sp. and ARGs in KOL18B3-3 and KOL18B3-10. *Bifidobacteria* sp. is an early intestinal colonizer and commensal which impacts the development of newborns in multiple beneficial ways and have been developed as probiotics for the prevention and treatment of diseases (25). Recent work by Bag et al. (26) and Duranti et al. (27) demonstrated the existence of ARGs in whole-genome sequence (WGS) of *Bifidobacteria* sp. Bag et al. showed the physical linkage of these ARGs against different classes of antimicrobials to MGEs in the WGS of a strain of *Bifidobacterium longum indica* that was isolated from feces of a healthy Indian adult (26). Duranti et al. found interspecies variation in breakpoint levels for antibiotics like streptomycin and tetracycline indicating the role of HGT in the evolution of AMR in *Bifidobacteria* sp. (27). Acquisition of ARGs by *Bifidobacteria* sp. has lead to its emergence as MDR and these in turn will serve as vehicles of transmission of ARGs.

This is the first study addressing the frequency and distribution of different classes of ARGs of diverse origin in the local intestinal milieu of diarrheal patients in Kolkata and the suburban areas. The structure and composition of microbiome is influenced by geography, ethnicity, and cultural practices. The study is distinct. It has been conducted using fecal samples from a discrete population. The study helped us to compare ARG profile between fecal samples from diarrheal patients afflicted with diarrhea due to different diarrheal pathogens. It helped us to conclude that fecal samples may share many ARGs in common, irrespective of the etiologic agent isolated from it. This is suggestive of a common gene pool acting as ARG reservoir and aiding in frequent lateral and vertical genetic exchange in pathogens and commensals. The study demonstrated that fecal samples from which routinely diagnosed pathogens were not isolated by conventional culture method also presented a resistome. These stool samples might have been harboring viral or eukaryotic diarrheal pathogens or any other enteric bacterial pathogen which was not tested for as it is beyond our scope. Further, it is difficult to obtain information from patients regarding self administration of antibiotics prior to their admission to the hospital. Self-administration of antibiotics is quite common in India due to over-the-counter sale of antibiotics and slack rules governing their purchase. The 16S rDNA sequence alignment from these fecal samples indicated that commensals and other members of the disturbed gut microflora or undiagnosed diarrheal agent may be contributing to the resistome. Although, metagenomics is the ultimate answer to the complex question about the structural and functional diversity of the microbiome, our findings obtained using common molecular methods is a highly significant pilot study indicating the presence and involvement of culturable and culture-resistant organisms in the development of AMR without the use of deep sequencing techniques and whole-genome sequence analysis. These findings are of immense importance for public health. It has enabled us to understand which resistant markers associated with different classes of antibiotics are present in the contemporary diarrheal microbiome and the relative abundance and frequency of the resistant markers will serve as parameters to understand and predict the potential threat that each of these classes of antibiotics will encounter leading to AMR crisis. Finally, our study will help in devising research strategies to intervene the transmission route of these genes to alleviate the problem of AMR.

## Data Availability Statement

All relevant data is contained within the article. All datasets generated for this study are included in the article/supplementary material.

## Ethics Statement

The study was conducted with the approval of the funding bodies and fecal samples were collected with the approval of the Institutional Ethics Committee, NICED. Informed consent was obtained from the participants of the study. Information obtained from the study participants was kept confidential. Information was kept locked in office. Any personal information was never passed to the third party.

## Author Contributions

RD planned, designed and executed experiments, analyzed the data, and wrote the paper. AM executed the experiments and analyzed the data. SD analyzed the data and wrote the paper.

### Conflict of Interest

The authors declare that the research was conducted in the absence of any commercial or financial relationships that could be construed as a potential conflict of interest.

## Footnotes

^1^https://www.who.int/news-room/fact-sheets/detail/antimicrobial-resistance

^2^www.clsi.org

## References

[1] Luo Y Luo R Ding H Ren X Luo H Zhang Y. Characterization of carbapenem-resistant *Escherichia coli* isolates through the whole-genome sequencing analysis. Microb Drug Resist. (2018) 24:175–80. 10.1089/mdr.2017.0079 28686503

[2] Hammerl JA Jäckel C Bortolaia V Schwartz K Bier N Hendriksen RS. Carbapenemase VCC-1–producing *Vibrio cholerae* in coastal waters of Germany. Emerg Infect Dis. (2017) 23:1735–37. 10.3201/eid2310.161625 28930017 PMC5621562

[3] Liu W Zou D Li Y Wang X He X Wei X. Sensitive and rapid detection of the New Delhi metallo-beta- lactamase gene by loop-mediated isothermal amplification. J Clin Microbiol. (2012) 50:1580–5. 10.1128/JCM.06647-11 22357496 PMC3347096

[4] Schmidt AS Bruun MS Larsen JL Dalsgaard I. Characterization of class 1 integrons associated with R-plasmids in clinical *Aeromonas salmonicida* isolates from various geographical areas. J Antimicrob Chemother. (2001) 47:735–43. 10.1093/jac/47.6.735 11389105

[5] Ceccarelli D Salvia AM Sami J Cappuccinelli P Colombo MM. New cluster of plasmid-located class 1 integrons in *Vibrio cholerae* O1 and a *dfrA15* cassette-containing integron in *Vibrio parahaemolyticus* isolated in Angola. Antimicrob Agents Chemother. (2006) 50:2493–99. 10.1128/AAC.01310-05 16801431 PMC1489794

[6] Hochhut B Lotfi Y Mazel D Faruque SM Woodgate R Waldor MK. Molecular analysis of antibiotic resistance gene clusters in *Vibrio cholerae* O139 and O1 SXT constins. Antimicrob Agents Chemother. (2001) 45:2991–3000. 10.1128/AAC.45.11.2991-3000.2001 11600347 PMC90773

[7] Iwanaga M Toma C Miyazato T Insisiengmay S Nakasone N Ehara M. Antibiotic resistance conferred by a class I integron and SXT constin in *Vibrio cholerae* O1 strains isolated in Laos. Antimicrob Agents Chemother. (2004) 48:2364–9. 10.1128/AAC.48.7.2364-2369.2004 15215082 PMC434172

[8] Nguyen MCP Woerther PL Bouvet M Andremont A Leclercq R Canu A *Escherichia coli* as reservoir for macrolide resistance genes. Emerg Infect Dis. (2009) 15:1648–50. 10.3201/eid1510.090696 19861064 PMC2866414

[9] Nonaka L Maruyama F Suzuki S Masuda M. Novel macrolide-resistance genes, *mef(C)* and *mph(G)*, carried by plasmids from *Vibrio* and *Photobacterium* isolated from sediment and seawater of a coastal aquaculture site. Lett Appl Microbiol. (2015) 61:1–6. 10.1111/lam.12414 25765542

[10] Dalsgaard A Forslund A Sandvang D Arntzen L Keddy K. *Vibrio cholerae* O1 outbreak isolates in Mozambique and South Africa in 1998 are multiple-drug resistant, contain the SXT element and the aadA2 gene located on class 1 integrons. J Antimicrob Chemother. (2001) 48:827–38. 10.1093/jac/48.6.827 11733467

[11] Guardabassi L Dijkshoorn L Collard JM Olsen JE Dalsgaard A. Distribution and *in-vitro* transfer of tetracycline resistance determinants in clinical and aquatic *Acinetobacter* strains. J Med Microbiol. (2000) 49:929–36. 10.1099/0022-1317-49-10-929 11023190

[12] Wang R Li J Kan B. Sequences of a co-existing SXT element, a chromosomal integron (CI) and an IncA/C plasmid and their roles in multidrug resistance in a *Vibrio cholerae* O1 El Tor strain. Int J Antimicrob Agents. (2016) 48:305–9. 10.1016/j.ijantimicag.2016.05.020 27470490

[13] Bag S Saha B Mehta O Anbumani D Kumar N Dayal M. An improved method for high quality metagenomics DNA extraction from human and environmental samples. Sci Rep. (2016) 6:26775. 10.1038/srep26775 27240745 PMC4886217

[14] Klindworth A Pruesse E Schweer T Peplies J Quast C Horn M. Evaluation of general 16S ribosomal RNA gene PCR primers for classical and next-generation sequencing-based diversity studies. Nucleic Acids Res. (2013) 41:e1. 10.1093/nar/gks808 22933715 PMC3592464

[15] Weisburg WG Barns SM Pelletier DA Lane DJ. 16S ribosomal DNA amplification for phylogenetic study. J Bacteriol. (1991) 173:697–703. 10.1128/jb.173.2.697-703.1991 1987160 PMC207061

[16] Wilson KH Blitchington RB Greene RC. Amplification of bacterial 16S ribosomal DNA with polymerase chain reaction. J Clin Microbiol. (1990) 28:1942–6. 2095137 10.1128/jcm.28.9.1942-1946.1990 PMC268083

[17] Wilson KH. Detection of culture-resistant bacterial pathogens by amplification and sequencing of ribosomal DNA. Clin Infect Dis. (1994) 18:958–62. 10.1093/clinids/18.6.958 7522060

[18] Stecher B. The roles of inflammation, nutrient availability and the commensal microbiota in enteric pathogen infection. Microbiol Spectr. (2015) 3:3. 10.1128/microbiolspec 26185088

[19] Andersen SR Sandaa RA. Distribution of tetracycline resistance determinants among gram-negative bacteria isolated from polluted and unpolluted marine sediments. Appl Environ Microbiol. (1994) 60:908–12. 8161183 10.1128/aem.60.3.908-912.1994 PMC201409

[20] DePaola A Roberts MC. Class D and E tetracycline resistance determinants in gram-negative bacteria from catfish ponds. Mol Cell Probes. (1995) 9:311–3. 8569770 10.1016/s0890-8508(95)91572-9

[21] Roberts MC. Tetracycline resistance determinants: mechanisms of action, regulation of expression, genetic mobility, and distribution. FEMS Microbiol Rev. (1996) 19:1–24. 10.1111/j.1574-6976.1996.tb00251.x 8916553

[22] Schmidt AS Bruun MS Dalsgaard I Larsen JL. Incidence distribution, and spread of tetracycline resistance determinants and integron-associated antibiotic resistance genes among motile *Aeromonads* from a fish farming environment. Appl Environ Microbiol. (2001) 67:5675–82. 10.1128/AEM.67.12.5675-5682.2001 11722922 PMC93359

[23] Stine OC Morris JG Jr. Circulation and transmission of clones of *Vibrio cholerae* during cholera outbreaks. Curr Top Microbiol Immunol. (2014) 379:181–93. 10.1007/82_2013_360 24407776 PMC4243509

[24] Faruque SM Nair GB Molecular Ecology of Toxigenic *Vibrio cholerae*. Microbiol Immunol. (2002) 46:59–66. 10.1111/j.1348-0421.2002.tb02659.x 11939579

[25] Hidalgo-Cantabrana C Delgado S Ruiz L Ruas-Madiedo P Sánchez B Margolles A. *Bifidobacteria* and their health-promoting effects. Microbiol Spec. (2017) 5:3. 10.1128/microbiolspec.BAD-0010-2016 28643627 PMC11687494

[26] Bag S Ghosh TS Banerjee S Mehta O Verma J Dayal M. Molecular insights into antimicrobial resistance traits of commensal human gut microbiota. Microb Ecol. (2019) 77:546–557. 10.1007/s00248-018-1228-7 30009332

[27] Duranti S Lugli GA Mancabelli L Turroni F Milani C Mangifesta M Prevalence of antibiotic resistance genes among human gut-derived Bifidobacteria. Appl Environ Microbiol. (2017) 17:83:e02894-16. 10.1128/AEM.02894-16 PMC5244288 27864179

