# Supplementary material for: Molecular Analysis of Selected Resistance Determinants in Diarrheal Fecal Samples Collected From Kolkata, India Reveals an Abundance of Resistance Genes and the Potential Role of the Microbiota in Its Dissemination
Source: Front Public Health. 2020 Mar 11;8:61. doi: 10.3389/fpubh.2020.00061 (PMC7078105; doi:10.3389/fpubh.2020.00061)
Supplement: Supplementary file 1 [file Table_1.DOCX]

1 2 3 4 5 6 7 L


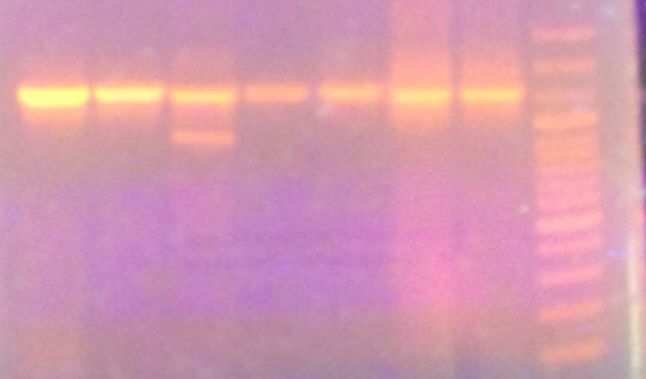


1500bp

**Figure S1**

Gel picture of PCR product obtained on amplification of 1500bp 16S rDNA fragment from C1-C9 region in representative samples. Lane1- KOL18B3-13, 2- KOL18B3-14, 3- KOL18B3-15, 4-KOL18B3-16, 5- KOL18B3-17, 6- KOL18B3-18, 7- N16961, L-1KB DNA Ladder
