# Supplementary material for: Molecular Analysis of Selected Resistance Determinants in Diarrheal Fecal Samples Collected From Kolkata, India Reveals an Abundance of Resistance Genes and the Potential Role of the Microbiota in Its Dissemination
Source: Front Public Health. 2020 Mar 11;8:61. doi: 10.3389/fpubh.2020.00061 (PMC7078105; doi:10.3389/fpubh.2020.00061)
Supplement: Supplementary file 4 [file Table_4.DOCX]

1 2 3 4 5 6 7 L


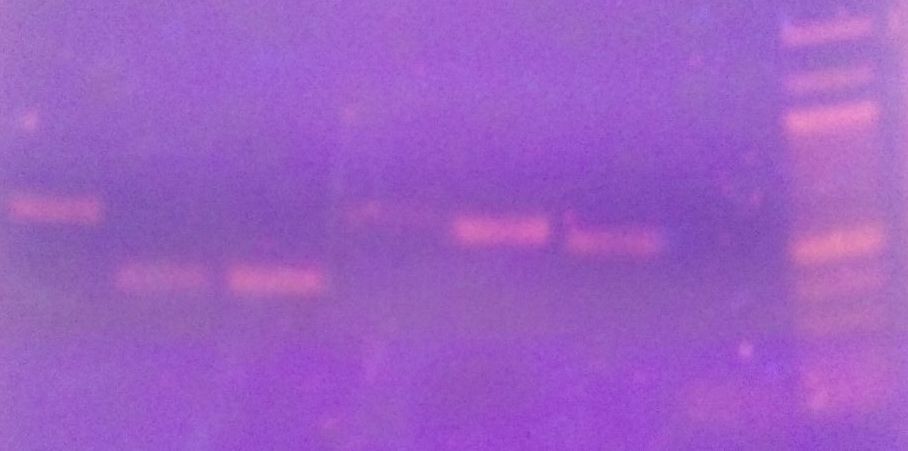


**Figure S4** Gel picture showing PCR amplification of ARGs. Lane 1-KOL18B2-3-*ant,* Lane 2*-*KOL18B2-2-*dhfr1,* Lane 3-KOL18B2-2-*aac3,* Lane 4-KOL18B2-3-*tetM,* Lane 5*-*KOL18B2-3-*tetC,* Lane 6-KOL18B2-3-*tnpA,* Lane 7*-* negative for all KOL18B3-13.
