# Supplementary material for: Molecular Analysis of Selected Resistance Determinants in Diarrheal Fecal Samples Collected From Kolkata, India Reveals an Abundance of Resistance Genes and the Potential Role of the Microbiota in Its Dissemination
Source: Front Public Health. 2020 Mar 11;8:61. doi: 10.3389/fpubh.2020.00061 (PMC7078105; doi:10.3389/fpubh.2020.00061)
Supplement: Supplementary file 5 [file Table_5.DOCX]

**S5** Assembled sequences of ARGs from representative fecal DNA to confirm the identity of the ARG whose presence was detected by PCR amplification

*cat1*

ACGTTCAGCTGGATATTACGGCCTTTTTAAGACCGTAAAGAAAAATAAGCACAAGTTTTATCCGGCCTTTATTCACATTCTTGCCCGCCTGATGAATGCTCATCCGGAATTCCGTATGGCAATGAAAGACGGTGAGCTGGTGATATGGGATAGTGTTCACCCTTGTTACACCGTTTTCCATGAGCAAACTGAAACGTTTTCATCGCTCTGGAGTGAATACCACGACGATTTCCGGCAGTTTCTACACATATATTCGCAAGATGTGGCGTGTTACGGTGAAAACCTGGCCTATTTCCCTAAAGGGTTTATTGAGAATATGTTTTTCGTCTCAGCCAATCCCTGGGTGAGTTTCACCAGTTTTGATTTAAACGTGGCCAATATGGACAACTTCTTCGCCCCCGTTTTCACCATGGGCAAATATTATACGCAAGGCGACAAGGTGCTGATGCCGCTGGCGATTCAGGTTCATCATGCCGTTTGTGATGGCTTCCATGTCGGCTGAATGCTTAATGAATTACAACAGTACTGCGAATGAGTGGCAGGGCCA

*tetC*

GCGACTCCTGCATTAGGAGCAGCCCAGTAGTAGGTTGAGGCCGTTGAGCACCGCCGCCGCAAGGAATGGTGCATGCAAGGAGATGGCGCCCAACAGTCCCCCGGCCACGGGGCCTGCCACCATACCCACGCCGAAACAAGCGCTCATGAGCCCGAAGTGGCGAGCCCGATCTTCCCCATCGGTGATGTCGGCGATATAGGCGCCAGCAACCGCACCTGTGGCGCCGGTGATGCCGGCCACGATGCGTCCGGCGTAGAGGATCCACAGGACGGGTGTGGTCGCCATGATCGCGTAGTCGATAGTGGCTCCAAGTAGCGAAGCGAGCAGGACTGGGCGGCGGCCAAAGCGGTCGGACAGTGCTCCGAGAACGGGTGCGCATAGAAATTGCATCAACGCATATAGCGCTAGCAGCACGCCATAGTGACTGGCGATGCTGTCGGAATGGACGATATCCCGCAAGAGGCCCGGCAGTACCGGCATAACCAAGCCTATGCCTACAGC

*tetM*

ACGATGACCTTTTAGAGAAATATATGTCCGGTAAATCATTAGAAGCATTGGAACTCGAACAAGAGGAAAGCATAAGATTTCAGAATTGTTCTCTGTTCCCTSTTTATCATGGAAGTGCAAAAAGTAATATAGGGATTGATAACCTTATAGAAGTTATTACTAATAAATTTTATTCATCAACACATCGAGGTCCGTCTGAACTTTGCGGAAATGTTTTCAAAATTGAATATACAAAAAAAAGACAACGTCTTGCATATATACGCCTTTATAGTGGAGTACTACATTTACGAGATTCGGTTAGAGTATCAGAAAAAGAAAAAATAAAAGTTACAGAAATGTATACTTCAATAAATGGTGAATTATGTAAGATTGATAGAGCTTATTCTGGAGAAATTGTTATTTTGCAAAATGAGTTTTTGAAGTTAAATAGTGTTCTTGGAGATACAAAACTATTGCCACAGAGAAAAAAGATTGAAAATCCGCACCCTCTACTACAAACAACTGTTGAACCGAGTAAACCTGAACAGAGAGAAATGTtGCT

*aadA1*

TTGTTGTGCACGACGACATCATTCCGTGGCGTTATCCAGCTAAA

*aadA1*

AGCGCGGAGAATCTCGCTCTCTCCAGGGGAAGCCGAAGTTTCCAAAACTC

*aminoglycoside N-acetyltransferase AAC(3)-IId*

CGTCACCCACCGTTTGTTGGGGATATCGGACAACCGCCGCGGCGATAGTGCAATGCCTTAACGGAGATTAGCGGCGCACCCAACAGCAGGGCCTTCCCGCCAAGGCGAACGAACCGCTCGACGGGCGATCCTTCCCCCAAGGCGTGACCGAGTTCGTGAG

*aminoglycoside 6'-N-acetyltransferase*

CTCGTAGCATCGCGATCGCTCGCAAGTTGCTCGGCGAGGAACCGTTTGGATCTTGGTGACCTCGGGATCATTGAACAGCAACCAACCAGAGCTCGAACCAGCTTGGTTCCCAAGCCTTTGCCCAGTTGTGATGCATTCGCCA

*Aminoglycoside 6'-N-acetyltransferase AAC(6')-Ib-cr5*

CGAGCTCTGGTTGAGTTGCTGTTCAATGATCCCGAGGTCACCAAGATCCAAACGGAACGACGCCGAGCAACTTGCGAGCGATCCGATGCTACGAGAAAGCGGGGTTTGAGAGGCAAGGTACCGTAACCACCCCATATGGTCCAGCCGTGTACATGGTT*A*

*aminoglycoside 3'-phosphotransferase*

CAATCTATCGATTGTATGGGAAGCCCGATGCGCCAGAGTTGTTTCTGAAACATGGCAAAGGTAGCGTTGCCAATGATGTTACAGATGAGATGGTCAGACTAAACTGGCTGACGGCATTTATGCCTCTTCCGACCATCAAGCATTTTATCCGTACTCCTGATGATGCATGGTTACTCACCACTGCGCTCCCCGGGAAAACAGCATTCCAGGTATTAGAAGAATATCCTGATTCAGGTGAAAATATTGTTGATGCGCTGGCAGCGTTCCTGCGCCGGTTGCATTCGATTCCTGTTAGTAATTGTCCTTTTAACAGCGATCGCGTATTTCGTCTCGCTCAGGCGCAATCACGAATGAATAACGGTTTGGTTGATGCGAGTGATTTTGATGACGAGCGTAATGGCTGGCCTGTTGAACAAGTCTGGAAAGAAATGCATAAGCTTTTGCCATTCTCACCGGA

*ANT(3'')-Ia family aminoglycoside nucleotidyltransferase*

CTTCAGTGATCTGCGCGTGAGGCCAAGTGATCTTCTTTTTGTCCCAGATAAGCTTGCTTAGAACAAGTAAGACGGGCTGATACTGGGCAGGTAGGCGTTTTAATGCCCAGTCGGCAGCGACATCCTTCGGCGCGATTTTGCCGGTTATTGCGCTGTACCAAATGCGGGACAACGTAAGCACTACATTTCGCTCATCGCCGGCCCAGTCGGGCTGCGAGTTCCATAGCTTCAAGGTTTCCCTCAGCGCCTCGAATAGATCCTGTTCAGGAACCGGGTCAAAGAATTCCTCCGCTGCCGGACCTACCAAGGCAACGCTATGTTCTCTTGCTTTTGTAAGCAGGATAGCTAGATCAATGTCGATCATGGCTGGCTCGAAGATACCCGCAAGAATGTCATTGCGCTGCCATTCTCCAAATTGCAGCTCGCGCTT

*DfrA1*

TATTTATGTTAGAGGCGAAGTCTTGGGTAAAAACTGGCCTAAAATTGCTGGGGATTTCAGGAAAGTAAACATCACCTTCCGGCTCGATGTCTATTGTAGATATATGTAGTGTATCTACTTGATCGATCAGGCTTTTGTATATCTCCCCACCACCTGAAACAATGAC

*dfrA12*

AGAAGGCGTCACCCTCGAAGGTTTGATGTACCTCAGAKAGaaACACGCCGTGGGCGTGAGGTAGTGCCAGAGTGTATATCTCAGCTCCGCCCGCGACGTAGAGTTCAGTGCCGAGTTCGGATGCCAAAGCGAT

*ereA*

AGCGATTTTCGGATACMCTGACCTTTTCTTTGTATGGCTCAGTGCTGATTTGGGTTAAATCATATCTACGCGAATCAGGAAGAAAACTGCAGTTAGTCGGAATCGATTTACCCAACACCTTGAATCCAAGGGACGACCTAGCGCAATTGGCCGAAATTATCCAGGTCATCGACCACCTCATGAAACCCCACGTTGATGCGCTGACTCAGTTGTTGACGTCCATTGATGGCCAGTCGGCGGTTATTTCATCGGCAAAATGGGGGGAGTTGGAAACGGCTCAGCAGGAGAAAGCTATCTCAGGGGTAACCAGATTGAAGCTCCGTTTGGCGTCGCTTGCCCCTGTCCTGAAAAATCACGTCAACAGCGATTTTTTCCGAAAAGCCTCTGATCGAATAGAGTC

IS110 family transposase CGATCTTTTTATGACCTGCTTTACTGCTTCCCTTTGATGTCATCCTACCTCCCGGGCTTGCCTGCCTAAGCCGACTGCCTGCCGCTCCCGCGGATAATAGGCTGCCGCTTTACCGCTTCCCGTCATTGATGACATTACAACTACATTACCATGATCTTGTTGAGACGATTGAAAAGTTGAAATCCCACTTGACACCCTCGTCGTCCTACGATGATCTTGGACCTGGCTTGCTGAATCTTCCCTGCGCCCGACCACAGACAGATAATGCTATTGCAACTGAAACTGTAAACGG

*intI2*

TGCACCATACAGCAGCGTAAAAATAACTTGGTTGTTAGTAGCCATAGGCTGAGAGTGAAGGAAGTTCATTTAAAAAGATAACCTAAGACATGGGCGTGCAAAGGTGGGGGGTTTTAATCCAACTTACCAAACGCAGAGTGTAACCATAAACACGCAAAAAATCGGTTTTTTCCCCAATTTTTTTGGCAACAGGGTTTACAACAGCCGGTGGGCGGATTGATTATATCCCTGGAGCAACCTCTACCGGACCCTCTGGTATCCCTTCAATGAAAGCAACCCATTTTGCAAGGATGGTACCCCCAACCAAATTATTTTTCCCCTTGGTGG

*intI1*

TACTTGCATTACAGCTTACGAACCGAACAGGCTTATGTCCACTGGGTTCGTGCCTTATAGGTTCCACGGTGTGCGTCACCCGGCAACCTTGGGCAGCAGCGAAGTCGAGGCATTTCTGTCCTGGCTGGCGAACGAGCGCAAGGTTTCGGTCTCCACGCATCGTCAGGCATTGGTTTTCTTTCGCTGTTCTTCTACGGCAAGGTGCTGTGCACGGATCTGCCCTGGCTTCAGGAGATCGGAAGACCTCGGCCGTCGCGGCGCTTGCCGGTGGTGCTGACCCCGGATGAAGTGGTTCGCATCCTCGGTTTTCTGGAAGGCGAGCATCGTTTGTTCGCCCAGCTTCTGTATGGAACGGGCATGCGGATCAGTGAGGGTTTGCAACTGCGGGTCAAGGATCTGGATTTCGATCACGGCACGATCATCGTGCGGGAGGGTTATTTTTTCCAAGGATCGGGCCTTGATGTTACCCGAGAGCTTGGCACCCAGCCTGCCCGAGCAGCTGTCGGGGGAACGGGCATGGTGGCTGAAGGACCAGGCCGAGGGCCGCAGCGGCGTTGCGTTTCCCGACGCCCTTTGGAGCGGAAGTATCCCCGCGCCGGGCATTCCTGGCCCGTGAATTCTGGGTTTTTGCCAAGCACAACAATTTCGACCAATACAACGGAGCGGGGGCGTGGCGTGCCCTTCCACTTGTATGAACCAGACCTTTCAGCGCGCCTTTCAACCGGGCCGAAAAAAAAAGCTGGTATTTACGATGCCCCGCCAAAGCCGGACGCCCTCCGCCCATTGGGATCGAGGAACGGGCGTTGGCTTACGCCAGCCGGGAAAGATAAAATTCCGAACACCGGTGGGAGGGACCCGGGCCTCGGCCCGATACCCGAACGGTCTCCTACCAAACAAATACATTTACACGCGCTATGTGCGCTGGAAAACGAATAGTAAA

*tetA*

GCCGGTGCTGCCGGGCCTCCTGCGCGATCTGGTTCACTCGAACGACGTCACCGCCCACTATGGCATTCTGCTGGCGCTGTATGCGTTGATGCAATTTGCCTGCGCACCTGTGCTGGGCGCGCTGTCGGATCGTTTCGGGCGGCGGCCGGTCTTGCTCGTCTCGCTGGCCGGCTCTGCTGACGACTACGCCATCATGGCGACGACGCCTTTCCTTTGGGTTC

*tetA*

CAGCAGGACCATGATCGGGAACGCCATCCATCCCCGTGTCGCGAAGGCAAGAAGGATGTAGCCTGTGCCGTCGGCAATCATTCCGAGCATGAGTGCCCGCCTTTCGCCGAGCCGGGCGGCTACAGGGCCGGTGATCATTGCCTGGGCGAGTGAATGCAGAATGCCAAATGCGGCAAGCGAAATGCCGATCGTGGTCGCGTCCCAGTGAAAGCGATCCTCGCCGAAAATGACCCAAAGCGCGGCCGGCACCTGTCCGACAAGTTGCATGATGAAGAAGACCGCCATCAGGGCGGCGACGACGGTCATGCCCCGGGCCCACCGGAACGAAGCGAGCGGGTTGAGAGCCTCCCGGCGTAACGGCCGGCGTTCGCCTTTGTGCGACTCCGG

*tetE*

TTATGCCTGTCTTGCCGGCGTTATTACGGGAGTTTGTTGGAAAGGCTAATGATGCAGAGAACTACGGTGTTTTATTGGCGCTGTATGCAATGATGCAAGTGATTTTTGCCCCTCTTCTCGGCCGCTGGTCAGATCGCATAGGTCGTCGCCCTGTATTGTTACTTTCACTTTCCTCCTGCAACACTGGACTACGCATTAATGGCAACAGCCAGCGTAGTGTGGGTGTTGTATTTGGGACGCTTAATTGCTGGTATTACCGGTGCGACTGGAGCTGTTGCAGCCTCAACAATTGCCGATGTCACACCTGAGGAATCCAGGACACATTGGTTTGGTATGATGGGTGCCTG

*mphA*

TGCATGCCGTCCCCATTTCCGCCGCCGTGGATGCGGGGATGCTCATCCGTACACCGACGCAGGCCCGTCAGAAGGTGGCCGACGACGTTGACCGCGTCCGACGCGAGTTCGTGGTGAACGACAAGCGCCTCCACCGGTGGCAGCGCTGGCTCGACGACGATTCGTCGTGGCCAGATTTCTCCGTGGTGGTGCATGGCGATCTCTACGTGGGCCATGTGCTCATCGACAACACGGAGCGCGTCAGCGGGATGATCGACTGGAGCGAGGCCCGCGTTGATG

*mefA*

GCTGTTCCATGCTACGGATAAACAATACTATCATAATCATCCAGACAGGTAGCTCCATACASAATGCAACAATAGCAAGCACTGCACCAGCTGCTGCGATAATTAAATCGGCACCAATCATTATCTTCTTCCTATCATGACGATCCACTARCACACCAATGGCAGGTCCCAAAATCGCATAGGGTAAAAAACCTACTAATGAAGCCATAGACAAGACCATCGCAGATCCTGTTttYTCTGTAAGGTAAAAAATrATCGCCATTTGCAGGATGGCACTAGTGT
