# Supplementary material for: Molecular Analysis of Selected Resistance Determinants in Diarrheal Fecal Samples Collected From Kolkata, India Reveals an Abundance of Resistance Genes and the Potential Role of the Microbiota in Its Dissemination
Source: Front Public Health. 2020 Mar 11;8:61. doi: 10.3389/fpubh.2020.00061 (PMC7078105; doi:10.3389/fpubh.2020.00061)
Supplement: Supplementary file 7 [file Table_7.DOCX]

**S7** Assembled sequences of 16S rDNA V3-V4 region 464bp amplicon

KOL18B2-1

TATCTAATCCTGTTTGCTCCCCACGCTTTCGCACCTGAGCGTCAGTCTTCGTCCAGGGGGCCGCCTTCGCCACCGGTATTCCTCCAGATCTCTACGCATTTCACCGCTACACCTGGAATTCTACCCCCCTCTACGAGACTCAAGCTTGCCAGTATCAGATGCAGTTCCCAGGTTGAGCCCGGGGATTTCACATCTGACTTAACAAACCGCCTGCGTGCGCTTTACGCCCAGTAATTCCGATTAACGCTTGCACCCTCCGTATTACCGCGGCTGCTGGCACGGAGTTAGCCGGTGCTTCTTCTGCGGGTAACGTCAATGAGCAAAGGTATTAACTTTACTCCCTTCCTCCCCGCT

KOL18B2-2

CCCATCACAAAAAGGACCGGCTAATTCCGTGCCAGCAGCCGCGGTAATACGGAAGGTCCGGGGGTTATCGGGATTTATTGGGTTTGACGGGAGCGAAGGGAGACCTTTAAGTTAACTGTGAAATACGGTTTCTCAACCGTCGAACTGCAGTTGATACTGGAGGTCTTGAGTGCACACTGGGATACTGGAATTCATGGAGTAGCGGAGGAATGCCTCTGATATCATGAAGAACTCCGATCGCGAAGTGAGGTCTACGGTCTGCAACTGACGCTGAGGCTCGAAAGTGATGCGTATCGGACACAGATTAGAT

KOL18B3-1

CTGCCTTCGCCATCGGAGTTCCTCCTGATATCTACGCATTTCACCGCTACACCAGGAATTCCACCTACCTCTTGCGCACTCAAGCCTGCCAGTTTCAAATGCAATTCCCAGGTTGAGCCCCGGGATTTCACATCTGACTTAACAAACCGCCTGCGCTCCCTTTACACCCAATAAATCCGGATAACGCTTGCACCCTACGTATTACCGCGGCTGCTGGCACGGAATTAGCCGGTGCTTATTCTTAAGGTACCGGCAA

KOL18B3-2

CACGCTTTCGCACCTGAGCGTCAGTCTTCGTCCAGGGaGGCCGCCTTCGCCACCGGTATTCCTCCAGATCTCTACGCATTTCACCGCTACACCTGGAATTCTACCCCCCTCTACGAGACTCAAGCTTGCCAGTATCAGATGCAGTTCCCAGGTTGAGCCCGGGGATTTCACATCTGACTTAACAAACCGCCTGCGTGCGCTTTACGCCCAGTAATTCCGATTAACGCTTGCACCCTCCGTATTACCGCGGCTGCTGGCACGGAGTTAGCCGGTGCTTCTTCTGCGGGTAACGTCAATGAGCAAAGGTATTAACTTTACTCCCTTCCTCCCCGCTGAAAGTACTTTACAACCCGAAGGCCTTCTTCATACACGCGGCATGGCTGCATCAGGCTtGCGCCCATTGTGCAATATTCCC

KOL18B3-3

CCGTTACACCGGGAATTCCAGTCTCCCCTACCGCACTCAAGCCCGCCCGTACCCGGTGCGGATCCACCGTTAAGCGATGGACTTTCACACCGGACGCGACTAACCGCCTACGAGCCCTTTACGCCCAATAATTCCGGATAACGCTTGCACCCTACGTATTACCGCGGCTGCTGG

KOL18B3-9

GCACCGGGTgATTCCTCCAtGATAtTCTACGCATTTCACCGCTACACMTGGGAATTCTACTCTCCTCTACTACACTCAAGCTTGACAGTATCAGATGCAGTTCCCAGGTTGAGCCCGGGGATTTCACATCTGATTTAACAAACCGCCTGCGTGCGCTTTA

KOL18B3-10

GCTTTCGCCATCTGGTGTTCTTCCAGATATCTACACATTCCACCGTTACACCGGGAATTCCAGTCTCCCCTACCGCACTCAAGCCCGCCCGTACCCGGCGCAAATCCACCGTATAAGACAGGGACTTTCACACCGACACGCGACAAACCGCCTACGAGCCCTTTACGCCCTTATTCCGGATAACGCTTGCACCCTACTAATTACCGCGGCTGCTGGCACGAATTTAGCCGGCGCTTATTCTAAAGGAACACTCCCGCGAAGGCTTGCTCCTCATACAAAGCGGCTTACAACCCAAAAGACCTCTTCCATCACGCGGCCTCCTTCACTCACGCTGGCT

KOL18B3-11

CTCAGCGTCAGTTACAGACAGAGAGCCGCTTTCGCCACCGGTGTTCCTCCATATATCTACGCATTTCACCGCTACACATGGAATTCCACTCTCCCCTTCTGCACTCAAGTTTGACAGTTTCCGAGCGAACTATGGGTGAGCCACAGCCTTTTCTTCCGACTTATCAAACCGCCTGCGCTCGCTTTTACCCCTAAATCCCGAAACGCTTTGGACCTACCTATTA

KOL18B3-11

TGACGAGCACGCCGCGTGAGTGATGAAGGTTCTTCGGATCTGTAAAGCTTGATGTAAGTCAAGAACGAGTGTGAGAGTGGAAAGTTCACACTGTGACGGTAGCTTACCAGAAAGGGACGGCTAACTACGTGCCAGCAGCCGCGGTAATACGTAGGTCCCGAGCGTTGTCCGGATTTATTGGGCGTAAAGCGAGCGCAGGCGGTTTGATAAGTCTGAAGTTAAAGGCTGTGGCTCAACCGTAGTTCGCTTTGGAAACTGTCAAACTTGAGTGCAGAAGGGGAGAGTGGAATTCCATGTGTAGCGGTGAAATGCGTAGATATATGGAGGAACACCGGTGGCGAAAGCGGCTCTCTGGTCTGTAACTGACGCTGAGGCTCGAAAGCGTGGGGAGCGAACAGGATTAGAT

KOL18B3-12

AATCCTGTTTGCTCCCCACGCTTTCGCACCTGAGCGTCAGTCTTCGTCCAGGGGGCCGCCTTCGCCACCGGTATTCCTCCAGATCTCTACGCATTTCACCGCTACACCTGGAATTCTACCCCCCTCTACGAGACTCAAGCTTGCCAGTATCAGATGCAGTTCCCAGGTTGAGCCCGGGGATTTCACATCTGACTTAACAAACCGCCTGCGTGCGCTTTACGCCCAGTAATTC-CGATTAACGCTTGCACCCT-CCGTATTACCGCGGCTGCTGGCACGGAGTTAGCCGGTGCTTCTTCTGCGGGTAcACGTCAgATGAGCAAAGGTATTAACTTTACTCCCTTCCTCCCtCGCTGAAAGTACTTTACAACCCGAAGGCCTTCTTCATACACGCGGCATGGCTGCATCAGgCTtGCGCCCATTGTGCAATATTCC

N16961

GCTaCaCCTGAAATtCTACCCCCcTCTaCAGTaCTCTAgCTTGTCAGTTCAAATgCGAtTCCTAGGTTGAGCCCAGGGCTTTCACATCTGACTTAACAAACCACCTGCATGCGCTTTACGCCCAGTAATTCCGATTAACGCTTGCACCCTCCGTATTACCGCGGCTGCTGGCACGGAGTTAGCCGGTGCTTCTTCTGTAGGTAACGTCAAATGATTAAGGTATTAaCTTAACCACCTTCCTCCCTACTGAAAGTACTTT

O395

AGGGAGGAAGGTGGTTAAGTTAATACCTTAATCATTTGACGTTACCTACAGAAGAAGCACCGGCTAACTCCGTGCCAGCAGCCGCGGTAATACGGAGGGTGCACAGATGTGAAAGCCCTGGGCTCAACCTAGGAATCGCATTTGAAACTGACAAGCTAGAGTACTGTAGAGGGGGGTAGAATTTCAGGTGTAGCGGTGAAATGCGTAGAGATCTGAAGGAATACCGGTGGCGAAGGCGGCCCCCTGGACAGATACTGACACTCAGATGCG
