# Supplementary material for: Molecular Analysis of Selected Resistance Determinants in Diarrheal Fecal Samples Collected From Kolkata, India Reveals an Abundance of Resistance Genes and the Potential Role of the Microbiota in Its Dissemination
Source: Front Public Health. 2020 Mar 11;8:61. doi: 10.3389/fpubh.2020.00061 (PMC7078105; doi:10.3389/fpubh.2020.00061)
Supplement: Supplementary file 8 [file Table_8.DOCX]

L 1 2 3 4 5 6 7 8 9 10 L


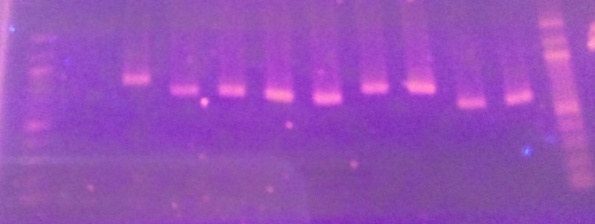


**Figure S8** PCR gel with amplified fragments of *gyrA, gyrB, parC, parE,*

*gyrA* Lane1- KOL18B2-1(negative) Lane2-KOL18B3-1

*gyrB* Lane 3- KOL18B2-1, Lane 4- KOL18B2-2, Lane 5-KOL18B3-1, Lane 6-KOL18B3-2

*parC* Lane 7-KOL18B2-2, Lane 8-KOL18B3-1

*parE* Lane 9-KOL18B2-2, Lane 10-KOL18B3-1
